# Combining two high-density QTL maps with a reference genome to identify candidate genes for morphology, yield, and biotic resistance in faba bean (*Vicia faba* L.)

**DOI:** 10.3389/fpls.2026.1832555

**Published:** 2026-04-29

**Authors:** Lorena Barea, Deepti Angra, Donal M. O’Sullivan, Carmen M. Ávila, Mariem Bouhadida, Ana M. Torres, Natalia Gutierrez

**Affiliations:** 1 Área de Mejora Vegetal y Biotecnología, Instituto de Investigación y Formación Agraria, Pesquera, Alimentaria y de la Producción Ecológica (IFAPA) Centro “Alameda del Obispo”, Córdoba, Spain; 2 Programa de Doctorado de Biociencias y Ciencias Agroalimentarias, Universidad de Córdoba, Córdoba, Spain; 3 School of Agriculture, Policy and Development, University of Reading, Reading, United Kingdom; 4 Field Crop Laboratory, National Institute for Agricultural Research of Tunisia (INRAT), University of Carthage, Rue Hédi Karray, Tunis, Tunisia

**Keywords:** biotic resistance, faba bean, high-density genetic map, morphology, physical map, quantitative trait loci (QTL), yield

## Abstract

Faba bean (*Vicia faba* L.) is a key legume crop for sustainable agriculture, but its improvement is constrained by yield instability and susceptibility to major biotic stresses. In this study, we developed high-density linkage maps for two recombinant inbred line (RIL) populations, 29H x Vf136 (P1) and Histal x L8 (P2), phenotyped across multiple environments and years, to identify stable QTLs associated with morphology, yield components and resistance to *Ascochyta fabae* and the parasitic weed *Orobanche* spp. Using the Vfaba_v2 Axiom SNP array, the maps comprised 2,043 and 3,903 SNPs for P1 and P2, respectively and were anchored to the reference genome to refine QTL intervals and prioritize candidate genes. Most traits exhibited high heritability and coherent phenotypic correlations, providing strong statistical power for QTL detection. A total of 59 QTLs were identified, with P1 QTLs primarily associated with biotic resistance and P2 QTLs encompassing morphological, yield and resistance to *A. fabae*. These high-density maps substantially improve resolution compared with previous studies, enabling refined QTL mapping, colocalization analyses and identification of biologically relevant candidate genes. Integration of these QTLs with previously reported QTL and GWAS data and projection onto the physical reference genome revealed 16 overlapping genomic regions (0.003 to 341.10 Mbp) containing 2 to 596 genes. The approach provided cross-validation and suggested the presence of stable loci consistently associated with these traits across diverse genetic backgrounds and environments. These overlapping regions were enriched in genes controlling development, productivity and biotic stress resistance and prioritized candidate genes included transcription factors (MADS-box, GATA, PLATZ, SAGA, CCCH zinc finger, WRKY, MYB-like), signalling proteins (protein kinases, LRR receptor-like proteins, Nudix hydrolases, calcineurin B-like, WD40), and growth and yield related enzymes (cytochrome P450s, glutathione S-transferases, glycosyltransferases, helicases, PPR, PUPs, PAPs, E3 ubiquitin ligases, RING finger, heparanase-like proteins). Several QTLs displayed potential pleiotropy, linking yield components with disease resistance traits. These findings provide a robust genomic framework for functional validation and marker-assisted breeding in faba bean, highlighting candidate genes and stable loci underlying complex agronomic traits.

## Introduction

Faba bean (*Vicia faba* L.) is one of the most important cool season legume crops, valued for its high yield potential and nutritional grains rich in protein, carbohydrate and fiber. It can also be used as animal feed and green manure, helping regulate soil health and improve fertility, key factors for the green and sustainable development of agriculture (Karkanis et al., 2018; Maalouf et al., 2018; Etemadi et al., 2019; Adhikari et al., 2021; Khazaei et al., 2021). Faba bean is among the most efficient nitrogen-fixing legumes and can be cultivated with little or no application of inorganic nitrogen fertilizer (Griffiths and Lawes, 1978; Baddeley et al., 2013; Singh et al., 2013). This contributes significantly to the sustainability of cropping systems and environmentally friendly agricultural practices. Despite these numerous advantages, global faba bean production remains lower than that of other major pulses such as common bean (*Phaseolus vulgaris* L.), chickpea (*Cicer arietinum* L.), field pea (*Pisum sativum* L.), cowpea (*Vigna unguiculata L*.), and lentil (*Lens culinaris* Medik.) (Adhikari et al., 2021).

According to the latest FAOSTAT (2023), faba bean is the sixth most produced legume crop globally. World production reached 1.69 Mt representing an increase of approximately 37% since 2000. China accounted for 27% of production followed by Ethiopia (19%), the United Kingdom (11%) and Australia (10%). Due to its adaptability to temperate and semi-arid climates, faba beans are cultivated throughout Europe, North Africa, the Middle East, and parts of Asia and Oceania. Although faba beans are gaining interest for sustainable agriculture, yield instability remains a major constraint. Production is frequently affected by biotic stresses including leaf diseases, parasitic weeds as well as abiotic stresses such as drought heat, and salinity (Adhikari et al., 2021). To overcome these challenges, current research is focusing on the development of more resilient cultivars and the optimization of agronomic practices to improve productivity and yield stability.

Among fungal diseases, ascochyta blight (*Ascochyta fabae*) and chocolate spot (*Botrytis fabae*) are two of the most devastating pathogens of faba bean in many regions including Europe, Canada, North Africa, Middle East and Oceania (Sillero et al., 2010; Atienza et al., 2016; Sudheesh et al., 2019). Parasitic weeds particularly *Orobanche crenata* and *Orobanche foetida* are root-parasitic species that can severely damage faba bean crops in Mediterranean Europe, West Asia and North Africa, often leading to drastic reductions in cultivated areas (Gressel et al., 2004; Maalouf et al., 2011; Rubiales et al., 2016; Boukteb et al., 2024). In addition, abiotic stresses such as heat, drought, frost and waterlogging further limit global crop productivity, with frost and waterlogging being more regionally specific (Arbaoui et al., 2008; Link et al., 2010). These combined biotic and abiotic constraints underscore the urgent need for integrated breeding strategies that combine field-based selection with molecular and genomic approaches to improve yield and environmental adaptability.

Faba bean is diploid (2n=12) and possesses one of the largest genomes among cultivated legumes (13,000 Mbp across six chromosomes) (Johnston et al., 1999), which has historically hindered genetic mapping and map-based cloning. While molecular breeding has advanced efficiently in crops like maize, rice, and wheat (Rao et al., 2014; Ordonio et al., 2016; Rodda et al., 2017), research in faba bean has long lagged behind. Progress has been accelerated by the recent availability of the faba bean reference genome (Jayakodi et al., 2023), allowing genomics to revolutionize faba bean breeding through detailed genome analysis.

Biparental mapping populations have been used in faba bean to develop genetic maps and to identify quantitative trait loci (QTL). These approaches allow breeders to identify and select genetic markers enhancing the efficiency and precision of breeding programs through techniques like Marker Assisted Selection (MAS). Early molecular maps, first with morphological, isoenzymes, random amplified polymorphic DNA (RAPD) and simple sequence repeats (SSR) provided an initial linkage framework (Torres et al., 1993; Satovic et al., 1996; Vaz Patto et al., 1999; Zeid et al., 2009; Ma et al., 2013). Subsequent consensus maps integrating SSR and single-nucleotide polymorphisms (SNPs) improved genome coverage and enabled orthologous (Ellwood et al., 2008) and cross-population comparisons (Satovic et al., 2013; Webb et al., 2016; Carrillo-Perdomo et al., 2020). The approach has allowed to identify QTLs associated with abiotic stress responses (Arbaoui et al., 2008) and biotic constraints including ascochyta blight (*A. fabae*) and broomrape (*O. crenata and O. foetida*) (Román et al., 2002, 2003; Avila et al., 2004, 2005; Díaz-Ruiz et al., 2009a, 2009b; Gutiérrez et al., 2013; Kaur et al., 2014; Atienza et al., 2016; Ocaña-Moral et al., 2017; Sudheesh et al., 2019; Gutierrez and Torres, 2021; Díaz-Ruiz et al., 2010) as well as morphological and yield-related traits (Cruz-Izquierdo et al., 2012; Ávila et al., 2017; Catt et al., 2017). Nevertheless, despite advances in mapping QTLs and identifying key genes, there are still challenges in applying this knowledge in agriculture.

The advent of high-throughput sequencing and genotyping platforms has further accelerated progress. At present, the use of a common high-density SNP genotyping array for both, GWAS in diversity panels and linkage mapping in biparental populations, allows better comparison of results, thereby strengthening the connection between these genetic mapping approaches. This standardized tool improves genome-wide coverage and helps bridge the gap from initial association signals detected in large populations to pinpointing putative candidate genes (Khazaei et al., 2021; Zhao et al., 2023; Gutierrez et al., 2024). Overall, the integration of high-density SNP maps, validated QTL intervals, and array-based GWAS provides a coherent framework for translating quantitative signals into functional candidates and deployable markers (Chen et al., 2025). This approach maintains continuity with earlier consensus maps and recent syntheses of genomic tools in faba bean (Satovic et al., 2013; Carrillo-Perdomo et al., 2020; Aguilar-Benitez et al., 2025).

Recent advances have demonstrated that dense SNP genotyping now delivers chromosome-scale resolution in faba beans. Using the V faba_v2 Axiom SNP array with 60K SNPs, high-density linkage maps have been developed and anchored to the *V. faba* reference genome, enabling fine-scale QTL delimitation and direct projection to physical coordinates (Khazaei et al., 2021; Jayakodi et al., 2023; Aguilar-Benitez et al., 2025). Mapping QTLs to the physical genome allows genetic intervals (cM, centimorgans) to be translated into chromosomal coordinates (Mbp, megabase pair), narrowing candidate regions and facilitating the identification of underlying genes. This anchoring, made possible by the reference genome and dense maps, also enables the integration of independent datasets such as GWAS and expression data, thereby accelerating the prioritization of targets for genetic improvement (Jayakodi et al., 2023; Mascher et al., 2024; Aguilar-Benitez et al., 2025; Gutierrez and Torres, 2025; Lippolis et al., 2025).

These advances have collectively transformed faba bean from a genomically underrepresented crop to one of the best characterised legumes at the chromosomal level. The combination of dense genetic maps, a high-quality reference genome and shared SNP platform now allows detailed analyses of the genetic basis of key agronomic traits and supports the implementation of efficient molecular breeding strategies.

In the present study, we therefore developed and compared two high-density genetic linkage maps constructed from recombinant inbred lines (RILs) derived from the crosses 29H × Vf136 (population 1) and Histal × L8 (population 2) using the Vfaba_v2 Axiom SNP array. QTL analyses were conducted for traits associated with morphology, yield and biotic resistance, enabling the identification of unique and shared genomic regions between populations. Furthermore, we integrated these QTLs with previously published QTL and GWAS results reported in the literature and projected the significant markers onto the physical faba bean reference genome to identify overlapping regions and prioritize candidate genes underlying key agronomic traits. Overall, these results provide new insights into the genetic architecture of complex traits and deliver molecular tools for marker-assisted selection and genomic selection in faba bean breeding.

## Materials and methods

### Plant material

Two RIL populations were used in this study. Population 1 (P1), segregating for resistance to broomrape (*O. crenata* and *O. foetida*) and *A. fabae*, was derived from the cross of the faba bean parental lines 29H and Vf136. This population consisted of 119 F_7:8_ individuals developed by the single seed descent method under insect proof cages. The genotype 29H, a minor type from INRA (France), is susceptible to broomrape (Gutiérrez et al., 2013) and resistant to *A. fabae* (Maurin and Tivoli, 1992; Sillero et al., 2001, 2010). The male parental Vf136, an equina type, was derived from the cross Vf1071 x Alameda at IFAPA (Cubero et al., 1992). This line shows resistance to broomrape but is susceptible to *A. fabae* (Román et al., 2003; Ávila et al., 2004; Díaz-Ruiz et al., 2009a, 2010; Gutierrez and Torres, 2021). Population 2 (P2), obtained from the cross Histal x L-831818 (hereafter L8), consists of 131 F_7:8_ individuals. Histal is a major variety developed by the seed company Semillas Fitó and is susceptible to *A. fabae*, whereas L8 is an equina IFAPA line highly resistant to this pathogen (Rubiales et al., 2012). Both populations segregate for a wide range of traits related to morphology, biotic stress resistance and yield-related traits.

### Phenotyping

Phenotypic evaluations of morphological, biotic stress resistance and yield related traits were carried out for both populations. Field trials were conducted in Córdoba, Spain (37.88° N, 4.78° W) during three growing seasons (2022/23, 2023/24, and 2024/25) and in Beja, Tunisia (36.73° N, 9.18° E) during 2019/20 and 2020/21, using a randomized design with two replicates per RIL population. Plants used for trait evaluation were randomly selected. Morphological and yield-related traits were measured and scored, following previously described methodologies to ensure consistency and comparability across experiments (Cruz-Izquierdo et al., 2012; Ávila et al., 2017; Aguilar-Benitez et al., 2022). For subsequent analyses, the phenotypic mean for each trait was used. Comprehensive descriptions of the evaluated traits, their corresponding acronyms, as well as details on the population and seasonal phenotypic assessments are provided in **Supplementary Table S1**.

#### Morphological traits

To evaluate the number of flowers per node (FN), five nodes were selected from each of three plants. For leaf size (LS), number of branches (NB) and plant height (PH), five plants per replicate were measured. To estimate pod length (PL) and seed size (SS), ten pods and ten seeds per RIL were selected. The leaves, pods and seeds were scanned, and the images were used to estimate the length of the pods and the area of the seeds and leaves. The open-source software “ImageJ” (Schneider et al., 2012), was used for this purpose.

#### Yield-related traits

Plot yield (PY) was determined as the total seed weight produced by each RIL expressed in grams (g). These seeds were subsequently used to determine the hundred seed weight (HSW) in grams (g). The number of pods per plant (PP), number of seeds per plant (SPL), pods per node in the field (PN), and shattering (SH) were recorded from five plants per replicate. The number of seeds per pod in the field (SP) was determined based on ten pods per RIL in each replicate.

#### Biotic resistance

##### Orobanche spp

The RIL population P1 (29H × Vf136), was evaluated for resistance to *O. foetida* under open field conditions in highly infested plots at Beja (Tunisia) during the 2019/20 and 2020/21 growing seasons. Ten seeds per RIL and parental lines were sown in rows following a randomized design with two replications, with each RIL flanked by two rows of the susceptible check cultivar ‘Bachar’, a Tunisian variety released by INRAT. Broomrape resistance for each RIL population was calculated as the number of emerged *O. foetida* shoots per faba bean plant, divided by the average number of emerged shoots in the flanking susceptible checks, following the methodology of Román et al. (2002). To correct for variability in field infestation, regression corrected values (residuals) were obtained using simple linear regression. The broomrape score of the susceptible checks was the independent variable, and the score of the RILs as the dependent variable. The lineal model was performed using the ‘lm’ function in the R ‘stats’ package, treating two replicates as a single blocking factor. An analysis of variance (ANOVA) was performed to test the significance of the blocking effect, and residual plots were examined to verify the criteria of normality and homoscedasticity. The residuals representing the difference between the observed values and predicted values were considered the broomrape resistance index. Finally, the broomrape resistance index value was range-standardized and transformed to allow comparison between years. This was done by subtracting the minimum index value from each observation and dividing by the total range of all values. The standardized broomrape resistance index was subtracted by 1 to assign higher values to more resistant lines, ranging from 0 (most susceptible) to 1 (most resistant).

Previous phenotypic evaluations of P1 for *O. crenata* (OC) at Córdoba (Spain) during the 2006/07, 2007/08, and 2008/09 growing seasons and at Kafr El-Sheikh (Egypt) in 2007/08, as well as for *O. foetida* at Beja (Tunisia) during the 2006/07 season, were incorporated in the present study to complete the resistance dataset (Díaz-Ruiz et al., 2009b; Gutiérrez et al., 2013).

##### Ascochyta fabae

Resistance to *A. fabae* was evaluated at the seedling stage under controlled growth chamber conditions in three experiments for P1 and P2, following the methodology described by Atienza et al. (2016). Five seeds of each accession were sown in 2L pots containing a 1:1 mixture of sand and peat, arranged in a randomized complete block design. Parental lines and susceptible checks were included in all evaluations.

Seedling resistance was assessed using the monoconidial isolate CO24, collected in Córdoba (Spain) in 2024 from naturally infected faba bean plants. The isolate was cultured on V8 medium for 14 days at 18 °C under a 12-h photoperiod. The resulting pycnidiospore suspension was adjusted to 1 × 10^6^ spores mL^−1^ using a hemocytometer. Inoculations were carried out when the fourth leaf was fully expanded by spraying the spore suspension until runoff. After inoculation, plants were maintained for 48 h in darkness at 100% relative humidity and then transferred to conditions of 18 °C with a photoperiod of 12 h light - 12 h dark. During the first week after inoculations, the plants were sprayed twice daily to ensure the presence of free water on the leaf surface which is essential for germination and penetration of the spores. The plants were irrigated three times per week.

Fifteen days after inoculation, disease resistance was scored on leaves (“disease severity on leaves”, DSL_ch) and stems (“disease severity on stems”, DSS_ch) and was based on the percentage of symptomatic area. Additionally, infection type (IT) was evaluated only in P1 using the 0–5 scale described by Rashid (1991).

Previous evaluations of *A. fabae* resistance in P1 were incorporated into the present study to support QTL analysis. These assessments included growth chamber (ch) conducted against two monocodial isolates, CO99 and LO98, from Córdoba and Logroño (Spain), respectively, as well as, field trial (F) evaluations performed in Córdoba during 2005–06 using a local isolate (Avila et al., 2004; Atienza et al., 2016).

### Statistical analysis

Descriptive statistics including mean, minimum and maximum value, standard deviation, coefficient of variation (CV) and heritability of the phenotypic data for all the evaluated traits in both populations were calculated using R 4.2.3 software (R Core Team, 2024). To investigate the relationship between traits, phenotypic correlations were estimated using Pearson’s correlation coefficient, implemented in the ‘PerformanceAnalytics’ R package (Peterson and Carl, 2024). Broad sense heritability (H^2^) was estimated using the Cullis method (Cullis et al., 2006) which is based on the average standard error of the genotypic Best Linear Unbiased Prediction (BLUPs) values. The ‘H2cal’ function from the ‘inti’ R package (Lozano-Isla, 2020), was used for this purpose. This function detects and filters outliers and fits a mixed model with genotype and replication as random effects. The broad-sense heritability was calculated according to the following equation:


$$H^{2}Cullis=1\;-(v\Delta BLUP/2*\sigma g2)$$

where “σ^2^g” is the genotypic variance, vΔ BLUP represents the average variance of the difference between genotypic BLUPs. In addition, a cluster analysis of observations was performed to find subgroups within mean phenotypic data in both populations by means of the K-means clustering method (Hartigan and Wong, 1979). The data were scaled to make variables comparable. The average silhouette approach was used to determine the optimal number of clusters. K-means clustering was performed using the built-in k-means libraries of R suite and similarities were measured by squared Euclidean distance to classify all the accessions into groups. The results were visualised by means of the ‘fviz_cluster’ function in the ‘factoextra’ R-package (Kassambara and Mundt, 2024).

### Genotyping and filtering

Young leaves from each genotype were collected, ground in liquid nitrogen, and used for total genomic DNA extraction using the DNeasy Plant Mini Kit (QIAGEN Ltd, UK) following the manufacturer’s instructions. DNA quality was evaluated by gel electrophoresis, and concentration was quantified using the QubitTM dsDNA BR Assay Kit (Invitrogen by ThermoFisher Scientific, UK). Genotyping was conducted using the Vfaba_v2 Axiom SNP array, comprising approximately 60,000 faba bean SNP markers (Khazaei et al., 2021). Segregating markers were filtered using the following quality criteria: call rate greater than 97%, minor allele frequency (MAF) greater than 30%, and heterozygosity less than 10%. RILs exhibiting poor DNA quality or more than 10% heterozygous loci were excluded from further analyses. Missing genotypes were imputed using the D-kNNi method (Money et al., 2015) implemented in Tassel v5.2.88 (Bradbury et al., 2007). Only SNP markers and RILs passing all quality control steps were retained for construction of the two high-density genetic linkage maps.

### Genetic Map construction

The genetics maps were constructed using the MAP algorithm in QTL IciMapping 4.2 software (Meng et al., 2015). Redundant markers were removed with the BIN algorithm using default parameters, and segregation data was assessed by chi-square tests against the expected 1:1 ratio. To determine the physical chromosomal positions of the SNP markers, their flanking sequences were aligned to the *V. faba* reference genome (Jayakodi et al., 2023) using the ‘map to reference’ tool in Geneious v.7.1.9 (Gutiérrez et al., 2023). Linkage groups (LGs) were defined using a heuristic LOD (logarithm of odds) threshold between 7 and 9 and marker ordering within each LG was performed with the mapping functions implemented in QTL IciMapping software. Recombination frequencies were converted into genetic distances (cM) using the Kosambi mapping function. The marker density along the linkage groups was visualized using the R package ‘ggplot2’ (Wickham, 2016), applying a 5 cM window.

### QTL mapping

QTL mapping in P1 and P2 was conducted using the high density genetic map and population-specific mean phenotypic data in the R package ‘R/qtl’ (v1.60) (Broman et al., 2003). QTLs were detected based on maximum LOD scores, with significance thresholds determined by 1,000 permutation tests (p < 0.05). Confidence intervals for each QTL were defined as the genomic regions flanking the peak LOD position within the 1-LOD for non-significant QTLs and 2-LOD support intervals for the significant QTLs. The percentage of phenotypic variance explained (R^2^) by each QTL was calculated following (Stansell et al., 2019). QTLs were named by combining the population acronym (P1 or P2), the trait abbreviation and the growing season of evaluation. The ID_QTL was used, to visualize QTL positions on the high density genetic maps, plotting the 1-LOD intervals (boxes) and 2-LOD intervals (bars) using the R package ‘LinkageMapView’ (Ouellette et al., 2018). The eggNOG-mapper v.2 with the eukaryotic database (Cantalapiedra et al., 2021) was used for functional annotation of the sequences flanking of each QTL associated marker.

### Integration of genetic and physical maps

A comparative assembly of genetic and physical maps for both RIL populations was generated to assess collinearity and validate marker order. This analysis was conducted and visualized using the Pretzel platform (Keeble-Gagnère et al., 2021); http://pulses.plantinformatics.io/mapview), which enabled the alignment of genetic linkage groups with their corresponding physical locations on the *Vicia faba* reference genome (Hedin v1). Pearson’s correlation analysis, conducted with the ‘Stats’ package in R, compared genetic distances (cM) with physical distances (Mbp) for each chromosome and population. This analysis quantifies the degree to which the marker arrangement on the genetic map (based on recombination frequencies), corresponded to their position on the physical map, allowing the identification of regions with local discrepancies, such as inversions in marker through deviations from the expected correlation.

### Defining overlapping regions and candidate genes identification

QTLs detected in P1 and P2 were anchored to the reference genome and projected onto the high-density physical map to detect overlapping regions with historical QTL mapping and GWAS analysis related with morphology, yield related traits and biotic resistance (Kaur et al., 2014; Sudheesh et al., 2019; Gutierrez and Torres, 2021; Skovbjerg et al., 2023; Zhao et al., 2023; Gutierrez et al., 2024; Ohm et al., 2024; Aguilar-Benitez et al., 2025; Lippolis et al., 2025). The criteria used to determine the overlapping regions (OR) were as follows: (1) For QTLs identified in the present study, the 1-LOD support interval was used to delimit the candidate region on the physical map; and (2) only previously reported QTLs and GWAS markers located within the confidence interval of the QTLs detected here were considered. The target interval of the each OR for the identification of the candidate genes associated with morphological traits, yield related traits and biotic stress resistance was defined as follows: (i) when an OR comprised exclusively overlapping QTLs, the confidence interval corresponded to the intersecting genomic regions shared by those QTLs, and (ii) when an OR resulted from the overlap between QTL and GWAS signals, the annotated genomic region was determined based on the local linkage disequilibrium (LD) decay previously reported (Gutierrez et al., 2024). The physical map information was used to estimate the confidence interval in Mbp and to determine the number of genes contained within each OR. Functional annotation of the candidate genes was done using eggNOG-mapper v.2 with the eukaryotic database (Cantalapiedra et al., 2021) based on fast orthology assignments using precomputed eggNOG v5.0 clusters and phylogenies (Huerta-Cepas et al., 2019). The QTLs detected in the present study (ID study 1) were visualized with the QTLs and GWAS described in previous studies (ID studies 2 to 10) on high-density map using the R ‘LinkageMap’ package (Ouellette et al., 2018), displaying the 1-LOD support intervals (QTLs) and linkage disequilibrium (GWAS).

## Results

### Field traits data

#### Population 1 (29H × Vf136)

Morphological traits (PH, LS, NB, PL, SS), exhibited moderate variation, with coefficients of variation (CV) ranging from 6.55 to 24.31% and high broad-sense heritability (H² = 0.73 - 0.97) (**Table 1**). Plant height (PH) increased slightly from a mean of 107 in 2024 to 116 cm in 2025, whereas leaf size (LS) declined from 59.59 cm² to 42.79 cm² over the same period. The number of branches per plant (NB) remained stable across years averaging approximately six branches per plant. Pod length (PL) and seed size (SS) showed low to moderate variability (CV = 8.72% and 14.09%, respectively) and very high heritability (H² = 0.96 and 0.97, respectively). Phenotypic correlations were significantly positive (**Supplementary Figure S1A**). Leaf size (LS) showed a moderate correlation with PL (r = 0.52) and PH (r = 0.45), indicating that taller plants tend to produce larger leaves and pods. Seed size (SS) also exhibited a moderated correlation with PL (r = 0.42).

**Table 1:** Descriptive statistics, proportion of phenotypic variation explained, and heritability of traits evaluated in two faba bean RIL populations (P1: 29H x Vf136 and P2: Histal x L8) across multiple years and environments including Córdoba and Logroño (Spain), Kafr El-Sheikh (Egypt) and Beja (Tunisia).

Population	Group of traits	Trait^a^	Environment	Year	N	Mean	Min	Max	SD	CV	V.g	V.e	V.p	H^2^
P1	Morphological	PH	Córdoba	2023/24	93	107.57	89.00	126.00	7.05	6.55	0.34	0.46	0.57	0.94
		PH	Córdoba	2024/25	93	116.20	101.00	138.50	7.75	6.67	0.24	0.53	0.51	0.90
		LS	Córdoba	2023/24	93	59.59	27.26	89.08	14.48	24.31	0.36	0.26	0.62	0.73
		LS	Córdoba	2024/25	93	42.79	29.04	58.71	6.09	14.24	0.21	0.50	0.46	0.90
		NB	Córdoba	2023/24	93	6.35	4.40	9.20	0.98	15.44	0.63	3.33	2.29	0.83
		NB	Córdoba	2024/25	93	6.42	3.80	8.30	0.89	13.92	0.11	0.81	0.52	0.78
		PL	Córdoba	2023/24	93	7.67	6.21	9.48	0.67	8.72	0.42	0.60	0.72	0.96
		SS	Córdoba	2023/24	93	0.83	0.54	1.22	0.12	14.09	0.01	0.01	0.02	0.97
	Yield	PY	Córdoba	2023/24	93	294.53	94.95	557.75	94.09	31.95	0.42	0.58	0.71	0.80
		HSW	Córdoba	2023/24	93	441.48	283.25	747.41	72.19	16.35	0.32	0.10	0.37	0.93
		SP	Córdoba	2023/24	91	3.63	3.10	4.55	0.30	8.30	0.08	0.34	0.25	0.91
		SH	Córdoba	2023/24	92	0.42	0.00	2.10	0.47	110.95	0.09	0.09	0.14	0.93
		SPL	Córdoba	2023/24	93	95.15	48.30	147.70	22.23	23.37	0.11	0.75	0.49	0.80
		PP	Córdoba	2023/24	93	36.78	16.80	63.00	8.80	23.94	0.15	0.78	0.54	0.83
	Biotic resistance	DSL_ch	Córdoba	1999	91	5.43	0.00	23.58	5.40	99.46	0.80	0.05	0.83	0.98
		DSL_ch	Logroño	1998	90	5.42	0.00	24.80	5.17	95.40	0.15	0.85	0.58	0.63
		DSL_ch	Córdoba	2024	93	6.13	0.00	26.83	5.35	87.37	0.78	0.19	0.88	0.96
		DSL_F	Córdoba	2005/06	90	14.31	0.00	42.50	12.30	86.01	0.69	0.28	0.83	0.91
		DSS_ch	Córdoba	1999	91	1.31	0.00	11.17	2.89	220.71	0.69	0.02	0.70	0.99
		DSS_ch	Logroño	1998	90	1.44	0.00	13.80	2.90	202.13	0.15	0.85	0.58	0.63
		DSS_ch	Córdoba	2024	93	2.26	0.00	16.00	3.32	147.09	0.29	0.11	0.34	0.94
		DSS_F	Córdoba	2005/06	90	6.39	0.00	25.00	5.98	93.56	0.29	0.24	0.41	0.84
		DSP_F	Córdoba	2005/06	90	2.91	0.00	4.00	1.02	35.17	0.56	0.41	0.76	0.86
		IT	Córdoba	2024	93	2.68	0.00	5.00	1.41	52.37	0.55	0.35	0.72	0.91
		Oc	Córdoba	2006/07	82	0.76	0.20	0.98	0.16	21.02	0.37	0.25	0.50	0.87
		Oc	Kafr El-Sheikh	2007/08	75	0.59	0.05	0.92	0.17	29.13	0.49	0.18	0.58	0.91
		Oc	Córdoba	2007/08	81	0.67	0.18	0.91	0.15	21.79	0.39	0.42	0.60	0.82
		Oc	Córdoba	2008/09	85	0.66	0.22	0.95	0.16	24.10	0.42	0.39	0.61	0.84
		Of	Beja	2006/07	89	0.66	0.04	0.96	0.17	25.83	0.08	0.25	0.20	0.68
		Of	Beja	2019/20	88	0.49	0.15	0.99	0.18	36.83	0.00	0.33	0.17	0.51
P2	Morphological	PH	Córdoba	2022/23	119	95.48	60.60	131.00	12.98	13.59	0.55	0.40	0.95	0.94
		PH	Córdoba	2024/25	119	120.74	100.00	141.00	7.73	6.40	0.34	0.61	0.65	0.76
		LS	Córdoba	2022/23	117	77.22	30.22	166.07	23.72	30.72	0.34	0.26	0.60	0.74
		LS	Córdoba	2024/25	116	107.35	67.74	145.32	15.30	14.25	0.08	0.76	0.46	0.58
		NB	Córdoba	2022/23	119	6.65	3.60	12.60	1.53	23.08	0.03	0.60	0.63	0.54
		NB	Córdoba	2024/25	119	4.91	3.00	7.00	0.78	15.79	0.22	0.78	0.61	0.68
		PL	Córdoba	2022/23	119	12.58	8.13	16.24	1.63	12.95	0.47	0.40	0.88	0.87
		SS	Córdoba	2022/23	118	2.06	1.11	3.34	0.41	20.09	0.08	0.08	0.16	0.54
		FN	Córdoba	2022/23	119	4.56	2.93	6.33	0.56	12.38	0.01	1.05	1.06	0.51
		FN	Córdoba	2024/25	118	4.27	3.03	5.23	0.41	9.58	0.15	0.85	0.57	0.63
	Yield	PY	Córdoba	2022/23	118	255.82	16.25	749.85	143.47	56.08	0.27	0.69	0.61	0.71
		PY	Córdoba	2024/25	119	192.29	14.20	550.61	111.76	58.12	0.11	0.83	0.53	0.60
		HSW	Córdoba	2022/23	118	93.80	38.75	136.94	20.28	21.62	0.62	0.26	0.75	0.91
		HSW	Córdoba	2024/25	118	107.74	53.66	174.48	22.03	20.45	0.16	0.58	0.45	0.67
		SP	Córdoba	2022/23	118	3.80	2.40	5.30	0.56	14.77	0.29	0.71	1.00	0.90
		SP	Córdoba	2024/25	119	3.97	2.45	5.40	0.52	13.07	0.46	0.49	0.71	0.82
		SPL	Córdoba	2024/25	119	34.77	7.50	90.40	15.28	43.94	0.26	0.74	0.63	0.70
		PP	Córdoba	2024/25	119	11.39	3.50	28.25	4.19	36.75	0.14	0.86	0.57	0.62
		PN	Córdoba	2022/23	118	1.07	0.27	1.93	0.33	30.94	0.07	0.64	0.70	0.79
	Biotic resistance	DSL_ch	Córdoba	2024	119	6.45	0.00	41.67	9.18	142.32	0.59	0.03	0.60	0.99
		DSS_ch	Córdoba	2024	119	3.08	0.00	27.33	5.32	172.89	0.33	0.09	0.37	0.94

N, number of observations; SD, standard deviation; CV, coefficient of variation; V.g, genotypic variation; V.e, environmental variance; V.p, phenotypic variance; H2, broad-sense heritability.

a PH, plant height; LS, leaf size; NB, number of branches per plant; PL, pod length; SS, seed size; FN, flowers per node measured in the field; PY, plot yield; HSW, hundred seed weight; SP, seeds per pod measured in the field; SPL, number of seeds per plant; PP, number of pods per plant; PN, pods per node measured in the field; SH, shattering; DSL_ch, Ascochyta fabae disease severity on leaves measured in chamber; DSS_ch, A. fabae disease severity on stems measured in growth chamber; DSL_F, A. fabae disease severity on leaves measured in the field; DSS_F, A. fabae disease severity on stems measured in the field; DSP_F, A. fabae disease severity on pods measured in the field; IT, A. fabae infection type measures on leaves; Oc, Orobanche crenata resistance; Of, Orobanche foetida resistance.

Yield-related traits (PY, HSW, SP, SH, SPL, PP) displayed the greatest phenotypic variability, (CV from 8.30% to 110.95%) and high heritability (H² from 0.80 to 0.93 (**Table 1**). Plot yield (PY) and hundred-seed weight (HSW) recorded in Córdoba in 2024, varied widely with PY ranging from 95 to 558 g and HSW from 283 to 747 g. Seeds per pod (SP) showed low variability (CV = 8.30%) and high heritability (H² = 0.91). In contrast, shattering (SH) exhibited very high variability (CV > 100%) but also high heritability (H² = 0.93). The number of seeds per plant (SPL) and pods per plant (PP) showed moderate variation (CV = 24%) and heritability between 0.80 and 0.83. Yield related traits displayed strong and significant inter-correlations (**Supplementary Figure S1B**). Plot yield (PY) was strongly associated with SPL (r = 0.79) and showed moderate to strong correlations with HSW (r = 0.67) and PP (r = 0.63), indicating that PY variation in this population is primarily driven by seed number and pods production. The strongest correlation among yield traits was observed between PP and SPL (r = 0.86), reflecting the expected biological relationship between the pod number and the total seed production.

Biotic resistance traits (DSL_ch, DSL_F, DSS_ch, DSS_F, DSP_F, IT, Oc, Of) exhibited the widest phenotypic variation, depending on the pathogen and the environment. For *A. fabae* (DSL_ch, DSL_F, DSS_ch, DSS_F, DSP_F, IT), evaluated in Córdoba in 1999, 2006 and 2024, the CV ranged from 35 to 220% with high and consistent heritability (H² = 0.63 - 0.99%) (**Table 1**). In contrast, resistance to *Orobanche* species, including *O. crenata* (Oc) and *O. foetida* (Of), showed moderate variability (CV = 22-37%) and moderate to high heritability (H² = 0.51-0.91) across locations and years (Córdoba 2007-2009; Beja 2007 and 2020; Egypt 2008). Correlation analysis among checks in the Of assay in Beja revealed inconsistency in the 2021 experiment; therefore, only the 2020 dataset was retained for subsequent analyses.

Resistance traits to *A. fabae* showed positive and consistent correlations across testing environments (chamber vs. field) and plant tissues evaluated (leaf vs. stem), supporting reliability of phenotypic selection for resistant genotypes (**Supplementary Figure S1C**). Leaf and stem disease severity measured in the chamber and in the field (DSL_ch; DSL_F and DSS_ch; DSS_F) were moderately correlated (r = 0.63 and 0.47, respectively). Correlations between leaf and stem severity were strong under both controlled (DSL_ch; DSS_ch; r = 0.85) and field conditions (DSL_F; DSS_F; r = 0.80). Disease severity in pods (DSP_F), measured only in the field, was moderately correlated with leaf and stem severity in the field (r = 0.50). The infection type scale (IT) exhibited moderate correlations with disease severity traits measured in both chamber and field conditions (≤ 0.64). In contrast broomrape resistance traits showed a different pattern with no significant phenotypic correlation between resistance to Oc and Of.

**Figure 1:**
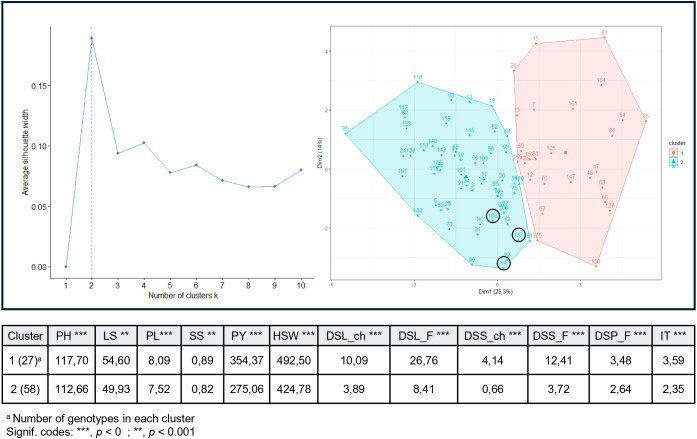
K-means clustering of 93 recombinant inbred lines (RILs) in population P1 (29H × Vf136) showing the optimal number of clusters based on average phenotypic trait values. Traits analyzed include PH, plant height; LS, leaf size; PL, pod length; SS, seed size; PY, plot yield; HSW, hundred seed weight; DSL_ch, *Ascochyta fabae* disease severity on leaves measured in chamber; DSL_F, *Ascochyta fabae* disease severity on leaves measured in the field; DSS_ch, *Ascochyta fabae* disease severity on stems measured in chamber; DSS_F, *Ascochyta fabae* disease severity on stems measured in the field; DSP_F, *Ascochyta fabae* disease severity on pods measured in the field; IT, *Ascochyta fabae* infection type on leaves. ^a^Number of genotypes in each cluster; Signif. codes: ***p < 0, **p < 0.001.

K-means clustering of RIL phenotypes for morphology, yield, and biotic resistance separate the lines in two clusters (**Figure 1**). Cluster 1 (27 genotypes) showed significantly higher average values (p < 0) for PH, PL, PY, HSW along with low resistance to *A. fabae*. Cluster 2 (58 genotypes) exhibited lower agronomic performance but maintained high resistance. Focusing on high-performing lines in Cluster 2, genotypes 54, 137 and 168 emerged as the most promising, combining moderate yield potential with robust *A. fabae* resistance.

#### Population 2 (Histal × L8)

Morphological traits (PH, LS, NB, PL, SS, FN) showed moderate variability, with CV values ranging from 6.4 to 30.7% and H² estimates from 0.51 to 0.94 (**Table 1**). Plant height (PH) ranged from 95.5 to 120.7 cm (CV = 6.4-13.6%), with higher mean values recorded in 2025. Leaf size (LS) exhibited the widest range (30.2–166 cm², CV = 30.7%), whereas the number of branches per plant (NB) was relatively stable (approximately 5 to 6 branches per plant) and exhibited low heritability (H² = 0.54 and 0.68). Pod length (PL) displayed low variability (CV = 12.9%) but high heritability (H² = 0.87) while seed size (SS) showed moderate variability (CV = 20%) with lower heritability (H² = 0.54). The number of flowers per node (FN) showed moderate variation (CV = 9.6-12.4%) with H² values ranging from 0.51 to 0.63. Significant positive correlations were detected between pod length (PL) and seed size (SS) (r = 0.67). Leaf size (LS) showed a weak positive correlation with PH (r = 0.33) (**Supplementary Figure S2A**).

Yield-related traits (PY, HSW, SP, SPL, PP, PN) displayed CV values ranging from 13.1% to 58.1% and H² estimates from 0.60 to 0.91 (**Table 1**). Plot yield (PY) varied widely across years, ranging from 16.2 to 750 g in 2023 and from 14.2 to 550.6 g in 2025, with high variability in both years (CV = 56% and 58%, respectively). Hundred-seed weight (HSW) remained relatively stable across years (≈ 100 g per 100 seeds, with moderate variability (CV = 20 – 22%) and moderate to high heritability (H² = 0.67-0.91). Seeds per pod (SP) exhibited low variability (CV = 13-14.8%) but high heritability (H² = 0.82-0.90). In contrast, the number of seeds per plant (SPL), pods per plant (PP) and pods per node (PN) showed higher variability (CV = 31-44%) with moderate heritability (H² = 0.62-0.79). Yield traits were moderately to strongly positively correlated (**Supplementary Figure S2B**). SPL and PP were the most strongly associated traits (r = 0.88), reflecting their joint contribution to yield. Plot yield (PY) correlated moderately with SPL (r = 0.51), HSW (r = 0.45), PP (r = 0.39) and SP (r = 0.31).

Biotic resistance traits corresponding to *A. fabae* (DSL_ch and DSS_ch), exhibited very high variability (CV = 142.3 - 172.9%), with high heritability (H² = 0.94-0.99), indicating a strong genetic component despite the wide phenotypic range (0-41.7 for DSL_ch and 0-27.3 for DSS_ch) (**Table 1**). Both traits (DSL_ch and DSS_ch) showed a strong positive correlation (r = 0.77) (**Supplementary Figure S2C**), suggesting that genotypes with higher leaf severity also tended to exhibit higher stem severity.

**Figure 2:**
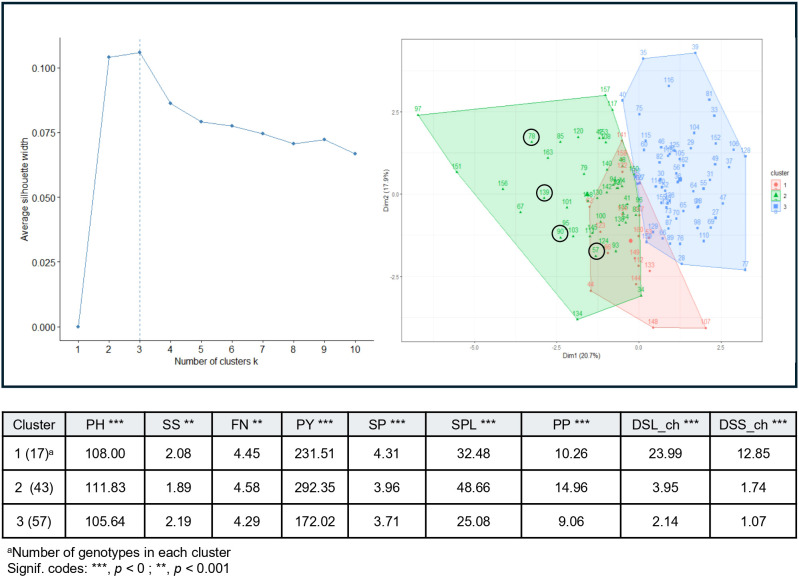
K-means clustering of 119 recombinant inbred lines (RILs) in population P2 (Histal × L8) showing the optimal number of clusters based on average phenotypic trait values. Traits analyzed: PPH,plant height; SS,seed size; FN,flowers per node in the field; PY,plot yield; SP,seed pod in the field; SPL,number of seeds per plant; PP, number of pods per plant; DSL_ch, *Ascochyta fabae* disease severity on leaves measured in chamber; DSS_ch, *Ascochyta fabae* disease severity on stems measured in chamber. ^a^Number of genotypes in each cluster; Signif. codes: ***p < 0, **p < 0.001.

K-means clustering of P2 revealed three groups (**Figure 2**). Cluster 1 (17 genotypes), comprised the most susceptible individuals, showing the highest disease severity for *A. fabae*. Cluster 2 (43 genotypes) included individuals with the highest mean values for PH, PY, SPL, PP along with moderate resistance. Cluster 3 (57 genotypes) contained the most resistant genotypes but showed the lowest values for morphological and yield traits. From Cluster 2, four genotypes (57, 78, 90 and 139) were selected as the best-performing lines combining high yield potential with minimal disease symptoms (**Figure 2**).

### Genotyping and linkage map construction

#### Population 1 (29H × Vf136)

**Figure 3:**
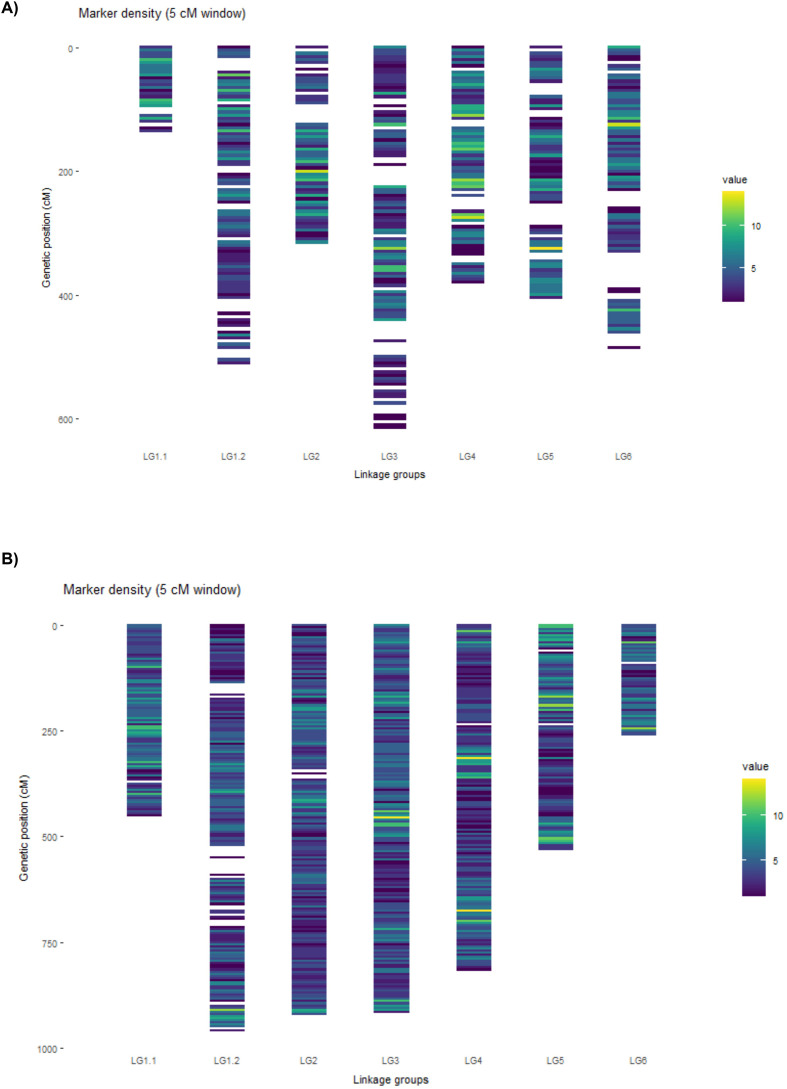
High-density genetic linkage maps of two faba bean RIL populations. **(A)** P1 (29H × Vf136). **(B)** P2 (Histal × L8). Linkage groups (LGs) are shown vertically and correspond to the six faba bean chromosomes. Marker positions are given in centimorgans (cM), and the shading scale represents marker density along each LG, calculated using a 5-cM sliding window.

Genotyping of 119 RILs and their parental lines using the Vfaba_v2 Axiom Array yielded 35,370 segregating polymorphic SNPs. Highly distorted markers that bias recombination estimates and markers order, were excluded to ensure an accurate and reliable genetic map After filtering of SNP markers and excluding individuals with more than 10% heterozygosity, 93 RILs were retained, resulting in a high-quality dataset comprising 3,635 SNP markers. Cluster analyses across a range of LOD thresholds indicated that values between 7 and 8.5 produced the most robust marker grouping, forming seven linkage groups (LGs) that correspond to the six faba bean chromosomes (**Figure 3A**; **Table 2**). Chromosome assignment of the SNP markers was performed by mapping the sequences against the faba bean genomic sequence (Jayakodi et al., 2023). Thus, from the 2,043 bin markers identified, 149 were unassigned (**Table 2**). The total map length was 2,860.27 cM (**Supplementary Table S2**), with an average of 292 bin markers per LG and a mean inter-marker distance of 1.37 cM (**Table 2**). Linkage group lengths ranged from 137.2 cM (LG1.1) to 616.36 cM (LG3), with a mean length of 408.61 cM. LG1.1 and LG 1.2 (chromosome 1) contained the highest number of bin markers (457), whereas LG2 had the fewest markers (261). The largest inter-marker gap was observed in LG6 (58,18 cM), while the smallest occurred in LG1.1 (13.35 cM). The chi-square test revealed that 9.7% of the bin markers exhibited distorted segregation with the highest numbers in LG3, LG4, LG5 and LG6 (33, 35, 35 and 59 respectively; **Table 2**).

**Table 2:** Information of the genetic maps developed in population 1 (P1, 29H x Vf136) and population 2 (P2, Histal x L8).

Population	Linkage group	No. of bin markers	No. of bin markers unassigned	Bin markers fitting 1:1 ratio	Bin markers outside 1:1 ratio	Total distance (cM)	Average distance (cM/bin marker)	Maximum gap (cM)
P1	1.1	128	8	126	2	137.20	1.08	13.35
	1.2	329	26	310	19	512.81	1.56	25.68
	2	261	10	245	16	318.35	1.22	31.47
	3	345	27	312	33	616.36	1.79	33.75
	4	361	31	326	35	381.14	1.06	23.06
	5	289	27	254	35	406.77	1.41	41.46
	6	330	20	271	59	487.63	1.48	58.18
	TOTAL	2,043	149	1,844	199	2,860.27		
P2	1.1	434	45	340	94	450.89	1.04	7.19
	1.2	625	99	541	84	961.67	1.54	44.13
	2	679	75	617	62	922.97	1.36	12.46
	3	795	111	604	191	918.91	1.16	7.58
	4	648	81	197	451	816.49	1.26	7.79
	5	474	83	433	41	532.31	1.13	10.62
	6	248	23	217	31	264.02	1.07	6.71
	**TOTAL**	**3,903**	**517**	**2,949**	**954**	**4,867.26**		

#### Population 2 (Histal × L8)

A total of 131 RIL individuals from P2 along with the parental lines were genotyped using the same high-density SNP array, obtaining 33,410 segregating SNP markers. Quality control led to the exclusion of 12 individual samples yielding a final dataset of 4,042 SNP markers. Grouping with LOD values between 7 and 9, produced seven LGs, which were assigned to the six chromosomes based on the reference genome (**Figure 3B**; **Table S2**). Of the total markers, 3,903 were grouped into bin markers, of which 517 could not be assigned to chromosomal position. The resulting genetic map spanned 4,867.26 cM (**Supplementary Table S3**) with an average of 558 bin markers per LG and a mean inter-marker distance of 1.22 cM (**Figure 3B**, **Table 2**). Linkage group lengths ranged from 264.02 cM (LG6) to 961.67 cM (LG1.2), with an average of 695 cM. LG3 contained the highest number of bin markers (795), whereas LG6 contained the fewest (248). The largest inter-marker gap was observed in LG1.2 (44.13 cM). The chi-square test revealed that 24.44% of the bin markers showed distorted segregation, with the highest numbers in LG4 and LG3 (451 and 191 respectively; **Table 2**).

### QTLs analyses

**Figure 4:**
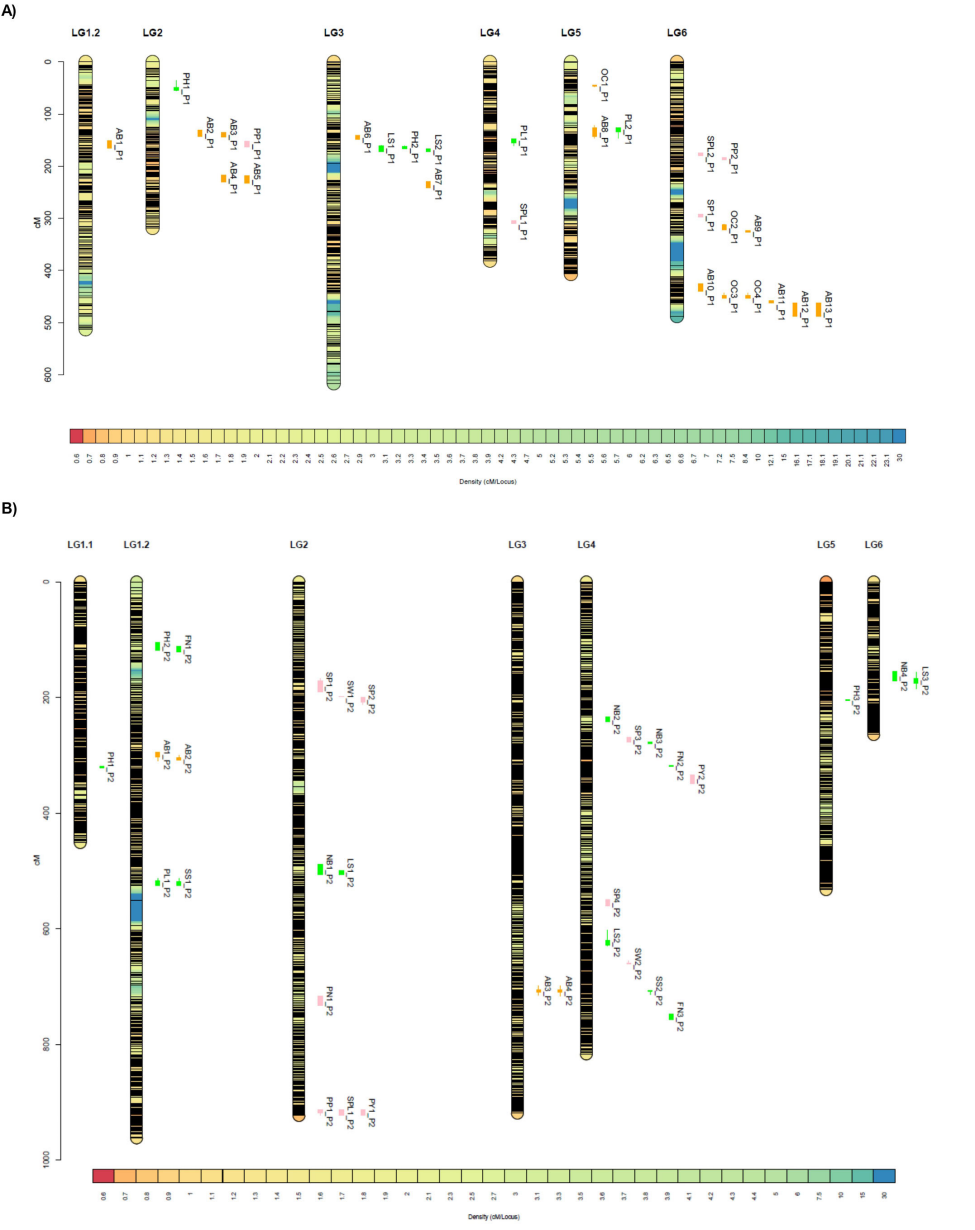
Distribution of QTLs on high-density genetic maps of two faba bean RIL populations. **(A)** P1 (29H × Vf136). **(B)** P2 (Histal × L8). QTLs are positioned along the linkage groups with boxes representing 1-LOD intervals, and bars representing 2-LOD intervals. QTLs are color-coded by trait category: morphology (green), yield-related traits (pink), and biotic resistance (orange). Traits abbreviations: AB, ascochyta blight; PH, plant height; PP, number of pods per plant; LS, leaf size; PL, pod length; SPL, number of seeds per plant; OC, *Orobanche crenata* resistance; SP, seeds per pod measured in the field; FN, flowers per node measured in the field; SS, seed size; SW, seed weight; NB, number of branches per plant; PN, pods per node measured in the field; PY, plot yield..

QTL mapping in the two faba bean populations revealed multiple genomic regions associated with morphological, disease and parasitic weed resistance and yield-related traits (**Table 3**). In population 1 (P1), analysis of 42 traits detected 28 QTLs, including six related to morphology, five to yield related traits and 17 to biotic resistance to O. crenata and *A. fabae* (no QTLs were detected for *O. foetida*). Of these, 20 did not reach the significance threshold but were retained for subsequent analyses (**Table 3**; **Supplementary Table S2**). In population 2 (P2), evaluation of 28 traits led to the identification of 31 QTLs comprising 16 for morphology, 11 for yield related traits and four for ascochyta resistance, eighteen of these were non-significant QTLs (**Supplementary Table S3**). High-density bin maps showing the distribution of both significant and non-significant QTLs are presented in **Figures 4A, B**.

**Table 3:** QTLs identified in P1 (29H x Vf136) and P2 (Histal x L8) using high-density genetic maps and corresponding phenotypic data.

Population	LG	ID	QTL name	Flanking markers (LOD -2)	Associated marker	Position associated marker (cM)	LODmax	LOD Threshold	R^2^ (%)	Description
P1	1.2	AB1_P1	DSS_ch_1999	AX-416824262/AX-181484889 (1g274440/1g290760)	AX-181199215 (1g274960)	154.00	1.96	3.14	9.25	GDP-L-galactose phosphorylase
	2	PH1_P1	PH_2024/25	AX-181468420/AX-181191782 (2g054120/2g064520)	AX-181147541 (2g058880)	51.80	4.21	3.18	18.82	Malonyl-CoA-acyl carrier protein transacylase
	2	AB2_P1	Af2_DSL_ch_1998	AX-181439532/AX-416787674 (2g049280/2g056200)	AX-181500890 (2g050640)	136.82	1.20	3.14	5.75	HATPase, heat shock
	2	AB3_P1	Af2_DSS_ch_1999	AX-416757457/AX-181154313 (2g046440/2g055120)	AX-181147798 (2g052680)	137.40	1.59	3.14	7.55	Heterogeneous nuclear ribonucleoprotein
	2	PP1_P1	PP_2023/24	AX-416759405/AX-181205299 (2g059880/2g070840)	AX-416747900 (2g067920)	159.00	1.98	3.20	9.34	-
	2	AB4_P1	Af2_DSL_ch_1999	AX-181168662/AX-416820010 (2g149240/2g169840)	AX-181486160 (2g159040)	220.81	1.81	3.14	8.55	kDa ribonucleoprotein
	2	AB5_P1	Af2_DSL_F_2005/06	AX-181481828/AX-181495149 (2g151760/2g170760)	AX-181462394 (2g158600)	220.81	1.97	3.14	9.29	Alcohol dehydrogenase-like
	3	AB6_P1	Af3_DSL_ch_2024	AX-181191513/AX-416791287 (3g090720/3g109720)	AX-416804920 (3g105080)	145.10	2.89	3.15	13.33	E3 ubiquitin-protein ligase
	3	PH2_P1	PH_2023/24	AX-181195713/AX-181462893 (3g150280/3g161640)	AX-416763051 (3g159640)	164.28	3.92	3.38	17.64	Domain of unknown function (DUF4228)
	3	LS1_P1	LS_2023/24	AX-416749511/AX-416760286 (3g143840/3g168680)	AX-416763051 (3g159640)	164.28	2.77	3.45	12.80	Domain of unknown function (DUF4228)
	3	LS2_P1	LS_2024/25	AX-181462893/AX-416760286 (3g161640/3g168680)	AX-416764557 (3g163520)	167.90	2.15	3.46	10.10	Serine threonine-protein kinase
	3	AB7_P1	DSS_ch_2024	AX-181443366/AX-416823821 (3g010560/3g017160)	AX-416761284 (3g012640)	232.70	2.75	2.93	12.72	3-ketoacyl-CoA synthase
	4	PL1_P1	PL_2023/24	AX-416823386/AX-181170246 (4g112200/4g123040)	AX-416812793 (4g115120)	153.96	5.34	3.46	23.24	RNA splicing
	4	SPL1_P1	SPL_2023/24	AX-181483910/AX-416803473 (4g176520/4g201960)	AX-181483162 (4g178560)	309.00	2.19	3.44	10.28	Galactinol--sucrose galactosyltransferase
	5	OC1_P1	Oc8_2006/07	AX-416808990/AX-416817831 (5g086680/5g090400)	AX-181168797 (5g084400)	45.50	3.56	3.36	16.16	Nudix hydrolase
	5	PL2_P1	PL_2023/24	AX-416806275/AX-181203152 (5g002200/5g008600)	AX-416806275 (5g002200)	125.66	3.46	3.46	15.75	DNA damage response protein
	5	AB8_P1	DSP3_F_2005/06	AX-181203152/AX-416825447 (5g008600/5g008960)	AX-181163039 (5g000360)	136.00	4.46	3.39	19.80	Chaperone protein
	6	SPL2_P1	SPL_2023/24	AX-181460393/AX-416814643 (6g138200/6g146680)	AX-181473091 (6g142840)	176.00	1.59	3.44	7.56	UDP-arabinose 4-epimerase
	6	PP2_P1	PP_2023/24	AX-181151511/AX-416722124 (6g147680/6g150880)	AX-416786837 (6g148400)	183.00	2.05	3.20	9.65	Sugar (and other) transporter
	6	SP1_P1	SP_2023/24	AX-181204620/AX-416802978 (6g153320/6g163320)	AX-181483331 (6g160560)	295.40	1.44	3.23	6.86	MACPF domain-containing protein At4g24290-like
	6	OC2_P1	Oc7_2008/09	AX-416759185/AX-416825877 (6g133560/6g144920)	AX-181147549 (6g136680)	316.90	3.45	3.31	15.70	Cyclic phosphodiesterase-like
	6	AB9_P1	IT_2024	AX-181457575/AX-416775010 (6g131800/6g134120)	AX-181462819 (6g132080)	325.91	2.24	4.14	10.50	Protochlorophyllide-dependent translocon component 52
	6	AB10_P1	DSP2_F_2005/06	AX-181445766/AX-416763058 (6g106640/6g128960)	AX-416737588 (6g106720)	426.21	2.82	3.39	13.05	Receptor-like serine threonine-protein kinase SD1-8
	6	OC3_P1	Oc7_2006/07	AX-416760602/AX-416789005 (6g131160/6g144360)	AX-416826116 (6g138880)	450.30	3.91	3.36	17.60	Assists the folding of proteins upon ATP hydrolysis
	6	OC4_P1	Oc7_2007/08	AX-416760602/AX-416789005 (6g131160/6g144360)	AX-416826116 (6g138880)	450.33	3.38	3.23	15.40	Assists the folding of proteins upon ATP hydrolysis
	6	AB11_P1	DSP1_F_2005/06	AX-416721640/AX-416750288 (6g153240/6g161720)	AX-416721640 (6g153240)	458.00	3.19	3.39	14.62	DNA-binding domain in plant proteins such as APETALA2 and EREBPs
	6	AB12_P1	DSL_ch_1998	AX-181178220/AX-416788273 (6g157560/-)	AX-416788273 (-)	487.63	1.27	3.14	6.11	-
	6	AB13_P1	DSL_ch_2024	AX-181178220/AX-416788273 (6g157560/-)	AX-416788273 (-)	487.63	3.10	3.14	14.23	-
P2	1.1	PH1_P2	PH_2024/25	AX-416796315/AX-416723588 (1g121000/1g124600)	AX-416796315 (1g121000)	318.91	2.27	3.52	8.40	Belongs to the glyceraldehyde-3-phosphate dehydrogenase family
	1.2	PH2_P2	PH_2022/23	AX-181459596/AX-181152418 (1g220680/1g228920)	AX-416758010 (1g220000)	109.18	3.37	3.63	12.23	-
	1.2	FN1_P2	FN_2024/25	AX-416770722/AX-416735502 (1g225760/1g225920)	AX-181152418 (1g228920)	118.70	2.33	3.62	8.63	Calcineurin-like phosphoesterase
	1.2	AB1_P2	DSS_2024	AX-181158093/AX-181190794 (1g288600/1g289600)	AX-416753560 (1g288800)	296.97	3.40	3.37	12.34	DNA repair and recombination protein
	1.2	AB2_P2	DSL_2024	AX-181470460/AX-416806768 (1g288640/1g289720)	AX-181173184 (1g289320)	306.60	5.12	3.44	17.98	Purine permease
	1.2	PL1_P2	PL_2022/23	AX-181486567/AX-416814529 (1g352800/1g358640)	AX-416746097 (1g353000)	524.00	5.06	3.67	17.78	Pentatricopeptide repeat-containing protein
	1.2	SS1_P2	SS_2022/23	AX-181486567/AX-416814529 (1g352800/1g358640)	AX-181486567 (1g352800)	524.70	4.75	3.43	16.79	Oxidative stress-related functions
	2	SP1_P2	SP_2022/23	AX-181496615/AX-181147710 (2g055640/2g058120)	AX-181147710 (2g058120)	190.80	4.34	3.58	15.46	28S ribosomal protein S29
	2	SW1_P2	HSW_2022/23	AX-416747037/AX-416767032 (2g060480/2g067800)	AX-416747037 (2g060480)	198.17	2.84	4.32	10.41	Ethylene-overproduction protein
	2	SP2_P2	SP_2024/25	AX-181441801/AX-416782365 (2g064800/2g068880)	AX-181441801 (2g064800)	199.10	3.80	3.44	13.69	Protein of unknown function (DUF1191)
	2	LS1_P2	LS_2024/25	AX-181491893/AX-181485180 (2g158640/2g160440)	AX-181471640 (2g161280)	505.00	3.05	3.60	11.13	Chromosome-associated kinesin
	2	NB1_P2	NB_2022/23	AX-416791867/AX-181485180 (2g148960/2g160440)	AX-181485180 (2g160440)	506.39	2.22	3.50	8.25	Tetratricopeptide repeat
	2	PN1_P2	PN_2022/23	AX-416732294/AX-416772340 (2g211280/2g212160)	AX-416815261 (2g217160)	730.50	3.30	3.65	11.99	Belongs to the glycosyl hydrolase 17 family
	2	PP1_P2	PP_2024/25	AX-416778408/AX-181179564 (2g274320/2g276400)	AX-416780918 (2g274160)	915.70	3.40	3.22	12.33	B-Box-type zinc finger
	2	SPL1_P2	SPL_2024/25	AX-416778408/AX-181179564 (2g274320/2g276400)	AX-416780918 (2g274160)	915.70	3.24	3.30	11.78	B-Box-type zinc finger
	2	PY1_P2	PY_2024/25	AX-416778408/AX-181179564 (2g274320/2g276400)	AX-416780918 (2g274160)	915.70	2.30	3.75	8.51	B-Box-type zinc finger
	3	AB3_P2	DSL_ch_2024	AX-181181163/AX-181484782 (3g182840/3g190080)	AX-416724138 (3g181640)	705.00	3.95	3.44	14.17	CST complex subunit
	3	AB4_P2	DSS_ch_2024	AX-181181163/AX-416743751 (3g182840/3g191680)	AX-416724138 (3g181640)	705.00	3.79	3.37	13.64	CST complex subunit
	4	NB2_P2	NB_2024/25	AX-181171234/AX-181165553 (4g050280/4g052040)	AX-181171234 (4g050280)	234.17	2.28	3.45	8.46	Nephrocystin-3-like
	4	SP3_P2	SP_2024/25	AX-416752040/AX-416813006 (4g052320/4g055600)	AX-416724237 (-)	270.71	2.26	3.44	8.38	-
	4	NB3_P2	NB_2022/23	AX-416813006/AX-416748860 (4g055600/4g057600)	AX-416813006 (4g055600)	277.50	2.27	3.50	8.42	Belongs to the eIF-2B alpha beta delta subunits family
	4	FN2_P2	FN_2022/23	AX-181448230/AX-181447622 (4g080520/4g092640)	AX-416774766 (4g069240)	318.10	2.69	3.54	9.89	-
	4	PY2_P2	PY_2022/23	AX-416803481/AX-416741304 (4g073840/4g084920)	AX-181177510 (4g101000)	337.80	3.14	3.47	11.44	-
	4	SP4_P2	SP_2022/23	AX-181164881/AX-416751822 (4g135000/4g142840)	AX-181164881 (4g135000)	549.20	3.49	3.58	12.62	Ras-related protein
	4	LS2_P2	LS_2024/25	AX-416740704/AX-416803949 (4g148160/4g156800)	AX-416821809 (4g154960)	623.00	4.36	3.60	15.53	LRR receptor-like serine threonine-protein kinase
	4	SW2_P2	SW_2022/23	AX-416768935/AX-181457061 (4g161120/4g167800)	AX-416725847 (4g163360)	660.00	6.77	4.32	23.05	-
	4	SS2_P2	SS_2022/23	AX-416812220/AX-416813503 (4g171840/4g208680)	AX-181472862 (4g176720)	707.50	5.53	3.43	19.27	TPR_17 superfamily Protein
	4	FN3_P2	FN_2022/23	AX-181447003/AX-416821316 (4g218080/4g221960)	AX-181443201 (4g220280)	750.06	1.91	3.54	7.13	Zinc Finger protein
	5	PH3_P2	PH_2024/25	AX-181203720/AX-416781902 (5g088840/5g089520)	AX-416765587 (5g088760)	203.40	2.51	3.52	9.26	Belongs to the Glu Leu Phe Val dehydrogenases family
	6	NB4_P2	NB_2024/25	AX-416729971/AX-416755798 (6g164960/6g168600)	AX-416772312 (6g166800)	163.20	2.54	3.45	9.36	Heavy-metal-associated domain
	6	LS3_P2	LS_2022/23	AX-181484674/AX-181173699 (6g164400/6g174920)	AX-416752492 (6g164600)	169.36	3.61	3.41	13.02	Belongs to the tRNA nucleotidyltransferase poly(A) polymerase family

a P1, Population 1: 29HxVf136; P2, Population 2: HistalxL8; AB, ascochyta blight; DSS_ch, Ascochyta fabae disease severity on stems measured in chamber; PH, plant height; PP, number of pods per plant; DSL_ch, *Ascochyta fabae* disease severity on leaves measured in chamber; LS, leaf size; IT, *Ascochyta fabae* infection type measures on leaves; PL, pod length; SPL, number of seeds per plant; OC, *Orobanche crenata* resistance; DSP_F, *Ascochyta fabae* disease severity on pods measured in the field; SP, seeds per pod measured in the field; FN, flowers per node measured in the field; SS, seed size; SW, seed weight; HSW, hundred seed weight; NB, number of branches per plant; PN, pods per node measured in the field; PY, plot yield.

In P1, QTLs were distributed across six linkage groups (**Table 3**; **Figure 4A**). On LG1.2, a single QTL for ascochyta resistance (AB1_P1) was identified, with the peak marker (AX-181199215) associated with a GDP-L-galactose phosphorylase. On LG2, a significant QTL for plant height (PH1_P1) was detected, whose peak marker (AX-181147541) corresponded to a malonyl-CoA-acyl carrier protein transacylase. Four QTLs related to *A. fabae* severity were identified and validated across different environments (chamber and field), plant tissues (leaf and stem) and years (Gutierrez and Torres, 2021). QTLs AB2_P1 and AB3_P1 located at ~136 cM, were linked to genes encoding an HATPase, heat shock–related protein and a heterogeneous nuclear ribonucleoprotein, respectively. QTLs AB4_P1 and AB5_P1, mapped at 220.81 cM and corresponded to a ribonucleoprotein and an alcohol dehydrogenase-like protein, respectively. In addition, a QTL for the number of pods per plant (PP1_P1) was detected at 159 cM. On LG3, two QTLs for *A. fabae* resistance (AB6_P1 and AB7_P1) were detected. AB6_P1 was associated with an E3 ubiquitin-protein ligase and AB7_P1 with a 3-ketoacyl-CoA synthase. AB6_P1 QTL corresponds with the Af3 (DSL/DSS) described by Gutierrez and Torres (2021). Three QTLs related to morphology were also detected: one for plant height (PH2_P1) and two for leaf size (LS1_P1 and LS2_P1). PH2_P1 and LS1_P1 co-localized and shared the same peak marker (AX-416763051) corresponding to a domain of unknown function (DUF4228). LS2_P1 mapped in proximity and was associated with a serine threonine-protein kinase. On LG4, a major QTL for pod length (PL1_P1) identified at 153.96 cM explaining a substantial proportion of the phenotypic variation (R^2^ = 23.24%) and associated with an RNA splicing-related protein. In a separate region (309 cM), QTL SPL1_P1 for the number of seeds per plant was detected, with the peak marker corresponding to a galactinol-sucrose galactosyltransferase. On LG5, two significant QTLs for biotic stress resistance were identified. OC1_P1 conferring resistance to *O. crenata*, previously identified as Oc8 by Gutierrez and Torres (2021), was linked to a Nudix hydrolase (AX-181168797) while AB8_P1, related to *A. fabae* severity in pods, corresponded to a chaperone protein (AX-181163039). Additionally, a QTL for pod length (PL2_P1) was detected and associated with a DNA damage response protein. LG6 harbored the highest number of QTLs, including three related to yield traits (SPL2_P1, PP2_P1 and SP1_P1) and eight associated with biotic stress resistance. The peak markers for SPL2_P1, PP2_P1 and SP1_P1 corresponded to an UDP-arabinose 4-epimerase, a sugar transporter and a MACPF domain-containing protein (At4g24290-like), respectively. A significant QTL for *O. crenata* resistance (OC2_P1), mapped at 316.9 cM with the peak marker associated with a cyclic phosphodiesterase-like in the same interval region of AB9_P1. A major QTL cluster was observed between 426.21 and 487.63 cM. Within this interval, AB10_P1, associated with *A. fabae* severity on pods, corresponded to a receptor-like serine threonine-protein kinase SD1-8. Two significant QTLs for *O. crenata* resistance (OC3_P1 and OC4_P1), validated in consecutive seasons, shared the same peak marker, corresponding to a protein involved in ATP-dependent protein folding. These co-localized with AB11_P1, associated with a DNA-binding domain protein. In the distal region, AB12_P1 and AB13_P1, both related to *A. fabae* severity on leaves, co-localized at the same peak marker (AX-416788273), for which no functional annotation is currently available (**Table 3**; **Figure 4A**). Notably, several biotic resistance QTLs on LG6 (OC2_P1, OC3_P1, OC4_P1, AB10_P1,AB11_P1, AB12 and AB13), were consistent with Oc7, DSL, DSP1 and DSP2 previously reported by Gutierrez and Torres (2021).

In P2, QTLs were distributed across all linkage groups (**Table 3**; **Figure 4B**). On LG1.1, a QTL for plant height (PH1_P2), was detected at 318.91 cM with the peak marker associated with a glyceraldehyde-3-phosphate dehydrogenase family protein. On LG1.2, PH2_P2 co-localized with FN1_P2, a QTL for flowers per node, associated with a calcineurin-like phosphoesterase. A second cluster at 296.97 to 306.60 cM included two significant QTLs for *A. fabae* severity: AB1_P2 on stems and AB2_P2 on leaves were consistent with AB1_P1 highlighting this interval as a conserved genomic region for ascochyta resistance between differents genetic background. Their peak markers corresponded to a DNA repair and recombination protein and a purine permease, respectively. In the distal region (~524 cM), two significant QTLs, PL1_P2 for pod length and SS1_P2 for seed size, overlapped and were associated with a pentatricopeptide repeat-containing protein and oxidative stress-related functions, respectively. On LG2, two significant QTLs for seeds per pod (SP1_P2 and SP2_P2) were identified linked to a 28S ribosomal protein S29 and a protein of unknown function (DUF1191), respectively. Both QTLs share the same genomic interval with PH_P1 and PP1_P1, identified in population 1, suggesting a conserved genomic region between different genetic backgrounds. Nearby, a non-significant QTL for hundred-seed weight (SW1_P2), corresponded to an ethylene-overproduction protein. QTLs for leaf size (LS1_P2, 505 cM) and branching (NB1_P2, 506.39 cM) were detected with peak markers corresponding to a chromosome-associated kinesin and a tetratricopeptide repeat protein, respectively. A QTL for pods per node (PN1_P2) was also identified and associated with a glycosyl hydrolase 17 family. Finally, a distal hotspot at ~915 cM included three QTLs related to yield components: pods per plant (PP1_P2), seeds per plant (SPL1_P2), and plot yield (PY1_P2). These QTLs shared the same peak marker (AX-416780918) corresponding to a B-Box-type zinc finger. On LG3, two significant QTLs for *A. fabae* severity on leaves and stems (AB3_P2 and AB4_P2) mapped to the same position (705 cM) and shared a peak marker associated with a CST complex subunit, proposing a novel genomic region involved in ascochyta blight resistance. LG4 contained two main QTL clusters. The first included QTLs for branching (NB2_P2 and NB3_P2) and flowers per node (FN2_P2), together with two yield related QTLs (SP3_P2 and PY2_P2). The second cluster included QTLs for seed weight (SW2_P2) and seed size (SS2_P2), which showed the strongest effects in P2 (R² = 23.05% and 19.27%, respectively). The SS2_P2 QTL overlaps the genomic region previously identified as SPL1_P1, highlighting its conservation among populations. These were associated with a protein of unknown function and to a TPR_17 superfamily protein. Additional QTLs in this LG were identified for seeds per pod (SP4_P2) and leaf size (LS2_P2), linked to a Ras-related protein and a LRR receptor-like serine threonine-protein kinase, respectively. On LG5, only one QTL for plant height (PH3_P2) was detected, associated with a dehydrogenase-family protein. Finally, on LG6, a QTL for branching (NB4_P2) and leaf size (LS3_P2) were identified, corresponding to a heavy-metal-associated domain protein and a tRNA nucleotidyltransferase poly(A) polymerase, respectively (**Table 3**; **Figure 4B**).

### Correlation of genetic and physical maps

In P1, correlations between genetic (cM) and physical (Mbp) marker positions varied markedly across linkage groups. Moderate to high correlations were observed for LG1/Vf1S (0.86), LG5/Vf5 (0.83), LG4/Vf4 (0.82), LG3/Vf3 (0.73) and LG6/Vf6 (0.63), while LG1/Vf1L showed the lowest correlation (0.29) (**Supplementary Figure S3**). Pretzel visualisations confirmed these patterns, showing highly aligned marker connections without detectable inversions or rearrangements. In contrast, P2 showed an almost perfect linear correspondence between the genetic and physical marker order with correlation coefficients ranging from 0.98 (LG4/Vf4) to 1.00 (LG2/Vf2 and LG6/Vf6) (**Supplementary Figure S4**).

### Overlapping regions and candidate genes identification

**Figure 5:**
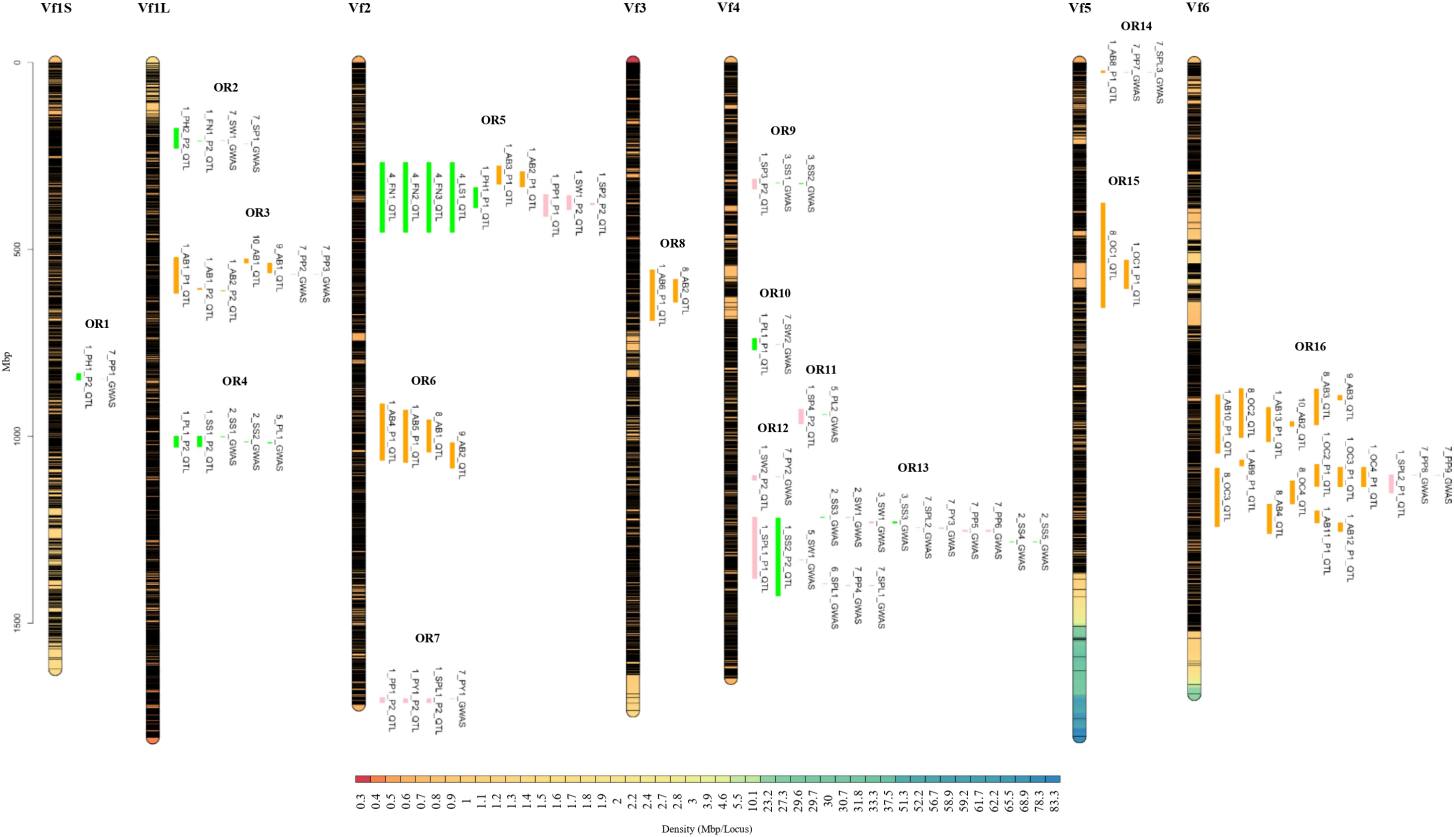
Physical genomic intervals defined by QTLs shared between faba bean RIL populations (ID study 1) and QTLs and GWAS signals from previous studies (ID study 2 to 10). QTLs and GWAS markers were projected onto the *Vicia faba* reference genome, identifying 16 overlapping regions (OR1-OR16) across the six faba bean chromosomes. Regions are color-coded by trait category: morphology (green), yield-related traits (pink), and biotic resistance (orange). Interval boundaries are based on the 1-LOD intervals for QTLs and linkage disequilibrium distances for GWAS markers, representing genomic regions used for putative candidate gene identification. Abbreviations: OR (overlapping regions); P1 (population 1, 29H x Vf136); P2 (population 2, Histal x L8); PP (pods per plant in the field); PH (plant height); FN (flowers per node in the field); SW (seed weight); SP (seed per pods); AB (Ascochyta blight); PL (pod length); SS (seed size); PY (plot yield); SPL (seeds per plant); OC (*Orobanche crenata* resistance).

Projection of QTLs onto the reference genome and integration of previously published QTL and GWAS data identified 16 overlapping regions (OR) across the six chromosomes (**Figure 5**; **Supplementary Table S4**). In these OR, QTLs detected in the present study (ID_Study 1) colocalized with previously reported loci associated with morphological, yield-related and biotic resistance traits (ID_Study 2 to 10). The overlapping intervals ranged from 0.003 to 341.10 Mbp and collectively harbored 2,288 genes, with each region containing between 2 and 596 genes (**Supplementary Table S4**).

On chromosome Vf1S, OR1, spans a narrow distance of 0.003 Mbp and contains 2 genes, including a plant height QTL (1_PH1_P2) and a GWAS signal for pods per plant (7_PP1_GWAS) reported by Gutierrez et al. (2024). Chromosome Vf1L contained three ORs. OR2 spans a 9.26 Mbp interval and contains 35 genes combining QTLs for plant height and flowers per node (1_PH2_P2, 1_FN1_P2) with GWAS for seed weight and seeds per pod (7_SW1_GWAS, 7_SP1_GWAS) previously reported by Gutierrez et al. (2024). OR3 (96.53 Mbp) contains 297 genes and clusters multiple ascochyta blight resistance QTLs detected in this study across different genetic backgrounds (1_AB1_P1, 1_AB1_P2, 1_AB2_P2). This region overlaps with previously reported resistance QTLs (10_AB1_QTL, 9_AB1_QTL) described by Sudheesh et al. (2019) and Kaur et al. (2014) as well as GWAS for pods per plant (7_PP2_GWAS, 7_PP3_GWAS) identified by Gutierrez et al. (2024). OR4 spans 17 Mbp and contains 53 genes linking QTLs for pod length and seed size QTLs (1_PL1_P2, 1_SS1_P2) with previously reported GWAS for seed size (2_SS1_GWAS, 2_SS2_GWAS; (Skovbjerg et al., 2023)) and pod length (5_PL1_GWAS; (Lippolis et al., 2025)) (**Figure 5**; **Supplementary Table S4**).

Chromosome Vf2 harbored three ORs. OR5 spans 135.31 Mbp and contains 425 genes, representing a major cluster integrating several QTLs detected in this study for plant height, ascochyta resistance, pods per plant, seed weight, and seeds per pod, detected in different genetic backgrounds (1_PH1_P1, 1_AB3_P1, 1_AB2_P1, 1_PP1_P1, 1_SW1_P2 and 1_SP2_P2). This region also overlaps with previously reported morphological QTLs associated with flowers per node and leaf size (4_FN1_QTL, 4_FN2_QTL, 4_FN3_QTL, 4_LS1_QTL) described by Aguilar-Benitez et al. (2025). OR6 spans 129.3 Mbp and contains 291 genes combining ascochyta resistance QTLs identified in this study (1_AB4_P1, 1_AB5_P1) with previous resistance QTLs (8_AB1_QTL and 9_AB2_QTL) described by Gutierrez and Torres (2021) and Kaur et al. (2014). Finally, OR7 (spanning 1.24 Mbp and containing eight genes), grouped yield-related QTLs (1_PP1_P2, 1_PY1_P2, 1_SPL1_P2) that overlap with a GWAS signal for plot yield (7_PY1_GWAS) reported by Gutierrez et al. (2024) (**Figure 5**; **Supplementary Table S4**).

On chromosome Vf3, one overlap region OR8 (159 genes with 62.11 Mbp of interval) where one QTL for ascochyta blight resistance detected in this study (1_AB6_P1) overlaps with Af3 (DSL/DSS) QTL for ascochyta blight resistance (8_AB2_QTL) reported by Gutierrez et al. (2024) (**Figure 5**; **Supplementary Table S4**).

Chromosome Vf4 comprises five ORs. >OR9 to OR12 (containing 6, 3, 5 and 6 genes, respectively) span narrow genomic intervals ranging from 0.18 to 2.59 Mbp. These regions represent individual overlaps between QTLs detected in this study and previously reported GWAS for seed size (3_SS1_GWAS, 3_SS2_GWAS), seed weight (7_SW2_GWAS), pod length (5_PL2_GWAS), and plot yield (7_PY2_GWAS), as described by Zhao et al. (2023); Gutierrez et al. (2024) and Lippolis et al. (2025). OR13 spans 183 Mb and contains 378 genes, representing a major hotspot where QTLs for seed size and seeds per plant (1_SS2_P2, 1_SPL1_P1) overlap with multiple GWAS associated with morphological and yield related traits. These GWAS signals were reported across several studies including six by Gutierrez et al. (2024), four by Skovbjerg et al. (2023), two by Zhao et al. (2023), one by Lippolis et al. (2025) and one by Ohm et al. (2024) (**Figure 5**; **Supplementary Table S4**).

Chromosome Vf5 contains two ORs. OR14 includes 7 genes within a narrow genomic interval, where an ascochyta blight resistance QTL (1_AB8_P1) overlaps with GWAS for pods and seeds per plant (7_PP7_GWAS, 7_SPL3_GWAS) reported by Gutierrez et al. (2024). OR15 spans 77 Mbp and contains 34 genes, including an *O. crenata* resistance QTL (1_OC1_P1) overlapping with a previously reported QTL (8_OC1_QTL) described by Gutierrez and Torres (2021) (**Figure 5**; **Supplementary Table S4**).

Finally, chromosome Vf6 includes a single large overlapping region, OR16 containing 596 genes within 341.1 Mbp genomic interval integrating multiple QTL associated with ascochyta blight and broomrape resistance. Several ascochyta blight resistance QTLs identified in this study (1_AB9_P1, 1_AB10_P1, 1_AB11_P1, 1_AB12_P1, 1_AB13_P1) overlap with resistance QTLs previously reported by Kaur et al. (2014); Sudheesh et al. (2019) and Gutierrez and Torres (2021). This region also contained broomrape resistance QTLs (1_OC2_P1, 1_OC3_P1, 1_OC4_P1) matching with previously reported QTLs from Gutierrez and Torres (2021), as well as a seeds per plant QTL (1_SPL2_P1) overlapping with GWAS for pods per plant (7_PP8_GWAS, 7_PP9_GWAS) reported by Gutierrez et al. (2024) (**Figure 5**; **Supplementary Table S4**).

The co-occurrence of genes involved in plant growth, morphology, yield related traits and stress responses within these intervals suggests that these regions may represent key regulatory hotspots influencing overall crop performance. To refine the candidate list, genes within each category were prioritized based on their functional annotations, previously reported roles in legumes and other crops or model species, and their biological relevance to the traits investigated in this study.

Across these ORs, numerous candidate genes with functions related to plant development, productivity, and defense responses were identified. Several transcription factor families known to regulate plant architecture and reproductive development were detected, including MADS-box, GATA, PLATZ, SAGA and CCCH zinc finger proteins. These regulators have been widely implicated in controlling flowering, seed development, plant architecture, and responses to environmental stimuli in legumes and other crop species. Genes involved in growth, metabolism, and yield formation were also prominent within the overlapping intervals. These included genes encoding protein kinases, cytochrome P450 enzymes, pentatricopeptide repeat proteins (PPR), glutathione S-transferases and glycosyltransferases. Additional candidates associated with seed size and yield components were identified, such as helicases, purine permeases, purple acid phosphatases, E3 ubiquitin-protein ligases, RING finger proteins, and heparanase-like proteins. Members of these gene families have been linked to seed development, nutrient allocation, and grain size regulation across diverse plant species.

In addition to developmental and yield-related genes, several loci associated with plant defense and stress responses were detected within the ORs. These included genes encoding NB-ARC domain proteins, Nudix hydrolases, NB-LRR (nucleotide-binding leucine-rich repeat) proteins, which are key components of plant immune signaling pathways. Transcription factors present in intervals included WRKY, MYB-like, and zinc finger proteins. Other defense-related candidates included calcineurin B-like proteins and WD40 repeat proteins, all of which have been implicated in pathogen recognition, signal transduction, and stress adaptation.

## Discussion

In this study, we developed high-density linkage maps for two faba bean RIL populations (P1 and P2), phenotyped in different environments and years to identify stable, colocalizing QTLs associated with morphology, yield, and biotic stress resistance. These genetic maps were aligned with the *V. faba* reference genome to anchor QTLs to physical coordinates and prioritise candidate genes within refined intervals.

Across both populations, biotic resistance traits, particularly those associated with *A. fabae*, showed greater phenotypic variability across years than morphological and yield traits. Nevertheless, most traits exhibited high broad sense heritability, indicating strong underlying genetic control. Phenotypic correlations were biologically coherent, thus developmentally or functionally related pod and seed traits were strongly positively correlated and disease severity in leaves correlated with severity in stems. This combination of wide phenotypic variation, high heritability and coherent trait correlations provided strong statistical power for QTL detection and candidate gene discovery. K-means clustering characterized phenotypic variation and helped identify genotypes combining favorable yield components with strong biotic resistance. Overall, these findings provide a solid foundation for selecting promising RILs and advancing marker-assisted breeding using the refined QTL intervals.

Using the Vfaba_v2 Axiom SNP array, we generated dense and well-resolved linkage maps for two faba bean RIL populations, providing the genomic resolution required for robust QTL detection in this large (~13 Gb) genome, where sparse and uneven marker coverage has previously limited genetic analyses (Khazaei et al., 2021). The final maps comprised 3,635 SNPs grouped in 2,043 bins for P1 and 4,042 SNPs in 3,903 bins for P2, comparable to other high-density maps developed using the same genotyping platform (Khazaei et al., 2021; Aguilar-Benitez et al., 2025). Although the maps were slightly less saturated than the ultra-dense map reported by Zhao et al. (2023), mean inter-marker distances of 1.37 cM (P1) and 1.22 cM (P2), provided sufficient resolution for QTL detection. The availability of the faba bean reference genome (Jayakodi et al., 2023) enabled consistent assignment of LGs to chromosomes and integration of previously unassigned markers.

Segregation distortion affected 9.74% of markers in P1 and 24.44% in P2; however, retaining these markers preserved recombination information, prevented artificially inflated map distances, and did not compromise QTL detection (Zuo et al., 2019). Although RILs populations of ~100 to 150 individuals may have limited power to detect small-effect QTLs (Alonso-Blanco et al., 2006), they are generally sufficient for detecting QTLs with moderate to large effects (Vales et al., 2005; Wang et al., 2012). With 93 individuals in P1 and 119 in P2, our populations fall within the recommended range for robust QTL mapping. Moreover, the clear assignment of the seven linkage groups to the six physical chromosomes further supports the quality of the genotyping data.

QTL mapping across the two RIL populations identified a moderate number of significant loci with effects that were consistent across traits, environments, and years, reflecting the genetics basis of agronomic performance and biotic resistance in faba bean. P1 yielded five significant QTLs mainly associated with resistance to *A. fabae* and *Orobanche* spp., with four QTLs for morphological and none for yield. In contrast P2 revealed 13 significant QTLs spanning morphological, pathogen plant resistance and yield-related traits, suggesting broader segregating variation and/or improved trait-resolution in this cross.

Comparison between genetic and physical maps (Keeble-Gagnère et al., 2019, 2021; http://pulses.plantinformatics.io/mapview) revealed marked differences in collinearity between the two populations (**Supplementary Figures S3**, **S4**). In P1, the low correlation observed in LG1/Vf1L indicates local inconsistencies in marker order, likely due to segregation distortion, heterogeneous recombination and population-specific genetic structure. In contrast, P2 showed almost complete collinearity across all linkage groups, supporting a highly consistent marker order and strong agreement between genetic and physical positions.

Overall, integrating the QTLs identified in this study with previously reported QTL and GWAS signals allowed the identification of 16 overlapping regions enriched in genes controlling plant development, productivity and resistance to biotic stresses (**Supplementary Table S4**; **Figure 5**). These regions likely represent key hubs for the genetic regulation of complex agronomic traits in faba bean and provide promising targets for future functional validation and marker-assisted breeding.

Among the possible regulators implicated in seed development, morphology and final yield, we have prioritized MADS-box, GATA, PLATZ, SAGA and CCCH zinc finger proteins. The MADS-box transcription factor plays a central role in crop growth, reproductive development and yield in different crops such as rice and maize (Adhikari and Kasahara, 2024; Wang et al., 2025). GATA proteins are strong candidates due to their involvement in regulating seed size, organ development, and stress tolerance in rice and *Arabidopsis* (Reyes et al., 2004; Richter et al., 2010). PLATZ factors regulate seed size and endosperm development across cereals and legumes (Li et al., 2017; Fu et al., 2020; Hu et al., 2023) while SAGA-associated factor 11 influences seed size (including seed width, seed length, and seed thickness), 100-seed weight, and total seed weight per plant in soybean (Liang et al., 2024a). Finally, CCCH zinc finger proteins contribute to crop yield primarily by enhancing abiotic stress tolerance, and modeling plant architecture (Yan et al., 2024; Zhao et al., 2024; Low et al., 2025).

Other key regulators detected were protein kinases, cytochrome P450 enzymes and pentatricopeptide repeat proteins (PPR), which coordinate plant growth, metabolism, yield formation and stress adaptation. Protein kinases in chickpea play key roles in pollen maturation, fertilization, flowering time and final pod and seed yield (Patil et al., 2024) and were already reported as promising candidates for faba bean yield related traits by Gutierrez et al. (2024) and Gutierrez and Torres (2025). In Arabidopsis, LRR receptor kinases such as IKU2, together with the WRKY transcription factor MINI3, regulate seed size (Luo et al., 2005; Li and Li, 2016) and allelic variation in the promoter of the a LRR-RLKs has been also implicated in melon seed size regulation (Liang et al., 2024b). Cytochrome P450 enzymes are known to positively regulate seed size and weight, in *Arabidopsis* and legume species such as soybean (Wang et al., 2015; Zhang et al., 2024) growth, development and responses to abiotic stresses in several legume species including *Medicago truncatula (*Song et al., 2017*;* Xia et al., 2021*)* and faba bean (Gutierrez and Torres, 2025). Pentatricopeptide repeat (PPR) proteins have likewise been associated with seed development and stress responses (Gutierrez et al., 2024; Wang and Tan, 2025). Additional candidate genes include glutathione S-transferases (GSTs) (Gong et al., 2005; Vaish et al., 2020) and glycosyltransferases. In cotton glycosyltransferases influence seed width and length, thereby affecting seed index and yield (Cao et al., 2024) whereas in rice, they enhance seed germination and modulates stress tolerance (Wang et al., 2020).

Additional candidates include helicases, purine permeases (PUPs) and purple acid phosphatases (PAPs). Helicases regulate stress responsive gene expression, contributing to plant defense and yield stability (Qi et al., 2012; Kanai et al., 2013; Mohapatra et al., 2023; Rehman et al., 2024) been reported in faba bean as potential candidates associated with yield traits such as seeds per pod and pods per plant across different environments (Gutierrez et al., 2024). Members of the PUP family, including Big Grain3 (bg3-D) in rice, have been identified as regulators of grain filling, grain weight, and seed setting rates (Xiao et al., 2019), while in lupin, they have been implicated in the transport of secondary metabolites (Martinez-Hernandez et al., 2025). Purple acid phosphatases have also been linked to plant development and productivity. In *Arabidopsis*, overexpression of a dual-targeted PAP leads to earlier bolting and significantly increased seed yield (Sun et al., 2012), while in soybean, overexpression of GmPAP14, although primarily involved in phosphorus uptake, promotes shoot biomass and improved plant growth (Kong et al., 2018). Finally, E3 ubiquitin-protein ligases, RING finger proteins and heparanase-like proteins are gene families linked to seed development, nutrient allocation, and grain size regulation across diverse plant species (Wang et al., 2015; Zhang et al., 2024). E3 ligases are key regulators of plant growth, development and stress responses (Shu and Yang, 2017; Shahwar et al., 2025) and have been associated with yield-related traits such as pods per plant in faba bean (Gutierrez et al., 2024; Gutierrez and Torres, 2025). RING finger proteins, which predominantly function as E3 ubiquitin ligases, also play major roles in seed yield; for example, Grain Width 2 (GW2) a RING-type E3 ligase in rice, regulates cell division and expansion (Ye et al., 2026), while ZmZFP2 a C4HC3-type RING finger protein in maize, controls cell division and kernel size (Zhang et al., 2025). Heparanase-like proteins have additionally been linked to major seed weight QTLs in soybean (Liang et al., 2024a).

In addition to developmental and yield-related genes, several genes associated with plant immunity and disease resistance were identified within the overlapping regions. NB-ARC and NB-LRR proteins, key components of resistance genes, recognize pathogen effectors and activate defence responses, and are widely distributed across model and crop species, including *Arabidopsis* and soybean (Wang et al., 2023). Similarly, receptor-like proteins (RLPs) perceive pathogen-associated molecular patterns and trigger immune signalling pathways in plants (Gao et al., 2025). Several transcription factors involved in defence regulation were also identified, including members of the WRKY, MYB-like and zinc finger protein families. WRKY transcription factors coordinate signalling pathways controlling defence gene expression across legumes and other crops (Song et al., 2018; Kankanala et al., 2019; Zhong et al., 2025), while MYB transcription factors such as the *Botrytis susceptible 1* (BOS) mediate jasmonate-dependent responses to biotic stress (Mengiste et al., 2003). Zinc finger proteins are similarly linked to stress tolerance and defence regulation in multiple crop species (Yan et al., 2024; Zhao et al., 2024; Low et al., 2025). Other potential candidate genes include calcineurin B-like proteins, WD40 repeat proteins and Nudix hydrolases, which participate in stress and hormone signalling. Calcineurin B-like proteins mediate stress and hormone pathways associated with defence (Meena et al., 2015), whereas WD40 proteins regulate hormonal and stress signalling pathways in species including *Arabidopsis*, rice and fruit crops (Li et al., 2025) and Nudix hydrolases such as AtNUDT7 act as regulators of defence responses, with mutations leading to enhanced disease resistance in *Arabidopsis* (Ge and Xia, 2008).

In faba bean, similar defence-related genes have been investigated through QTL fine-mapping approaches. Gutierrez and Torres (2021) analysed two RIL populations (29H × Vf136 and Vf6 × Vf136) using synteny with *Medicago truncatula* to identify candidate genes within QTLs for resistance to *Ascochyta fabae* and *Orobanche crenata*. A total of 219 defence-related candidate genes were screened in these regions, including 30 WRKY transcription factors, 21 NBS-LRR resistance gene analogues, four zinc finger proteins, three MYB transcription factors and two WD repeat proteins. Together, these results highlight the potential importance of these genomic regions in regulating biotic stress resistance in faba bean.

QTLs and GWAS signals identified in this, and other studies shared candidate genes within the same genomic regions, providing cross-validation and suggesting the presence of stable loci consistently associated with the specific traits across diverse genetic backgrounds and environments. Relevant examples include Nudix hydrolases, E3 ubiquitin-protein ligases, Serine threonine-protein kinases, purine permeases, pentatricopeptide repeat-containing protein, LRR receptor-like proteins and zinc finger protein whose QTL LOD peaks felt within the confidence intervals of related traits, reported in multiple studies (Gutierrez and Torres, 2021; Gutierrez et al., 2024; Patil et al., 2024; Gutierrez and Torres, 2025; Gao et al., 2025). This pattern was particularly evident in OR3, OR6, OR8 where QTLs for ascochyta resistance detected in the present study colocalized between P1 and P2 and overlapped with previously reported QTLs by Kaur et al. (2014); Sudheesh et al. (2019) and Gutierrez and Torres (2021). In OR3 and OR14, overlapping QTLs for *Ascochyta* resistance (AB) and previous GWAS for pods per plant (PP) and seeds per plant (SPL) (Gutierrez et al., 2024), suggest potential pleiotropic effects or tight linkage between defense and yield components. Similarly, co-localization was observed in OR16 *A. fabae* and *O. crenata* resistance QTLs detected here and in previous studies (Kaur et al., 2014; Sudheesh et al., 2019; Gutierrez and Torres, 2021). In OR7, QTLs for plant yield (PY) identified here overlapped with GWAS signals reported by Gutierrez et al. (2024). Furthermore, convergence between QTL mapping and GWAS for yield related traits was observed in OR4 (seed size and pod length), OR7 (plant yield), and OR13 (seed size, seed weight, seeds per plant and pods per plant), providing independent validation of these genomic regions. This overlap strengthens the evidence supporting the underlying candidate genes and highlights their potential as targets for pyramiding high yield and disease resistance. Further functional validation and fine-mapping studies are required to confirm candidate genes and clarify their roles in trait variation. Additionally, the application of genomic prediction models could help estimate breeding values for multiple traits simultaneously, facilitating more efficient selection in breeding programs.

## Conclusions

This study developed and compared high-density genetic maps for two *V. faba* RIL populations using the Vfaba_v2 Axiom SNP array. These maps represent a substantial improvement over previous studies, providing the resolution needed to dissect complex traits related to morphology, yield and biotic resistance. The projection of the QTLs detected with historical QTLs and GWAs analysis related with these traits onto the high-density physical faba bean map validated marker collinearity, anchored QTL intervals to the reference genome and identified and validated shared genomic regions across populations, providing a robust framework for candidate gene discovery. Sixteen stable overlapping intervals were detected across populations and environments and the colocalized genes within these intervals highlight promising candidate loci for *A. fabae* resistance and yield improvement. Collectively, these high-density maps and prioritized candidate genes provide valuable resources for marker assisted selection and a strong foundation for future functional validation in faba bean.

## Data Availability

The datasets analyzed for this study can be found in the online repository Figshare. This data can be found here: https://doi.org/10.6084/m9.figshare.31064770.
