# Supplementary material for: Combining two high-density QTL maps with a reference genome to identify candidate genes for morphology, yield, and biotic resistance in faba bean (Vicia faba L.)
Source: Front Plant Sci. 2026 Apr 29;17:1832555. doi: 10.3389/fpls.2026.1832555 (PMC13167940; doi:10.3389/fpls.2026.1832555)

Supplementary Figure S2. **Pearson correlations and histograms showing the distribution of traits in RIL population 2 (Histal x L8).** A) Phenotypic frequency and correlation between plant morphology traits: PH (plant height), LS (leaf size); NB (number of branches measured in the field); PL (pod length); SS (seed size); FN (flowers per node measured in the field). B) Phenotypic frequency and correlation between yield-related traits: PY (plot yield); HSW (hundred seed weight); SP (seeds per pod measured in the field); SPL (number of seeds per plant); PP (number of pods per plant); PN (pods per node measured in the field). C) Phenotypic frequency and correlation between disease resistance traits: DSL_ch (Ascochyta. fabae disease severity on leaves measured in chamber) ; DSS_ch (A. fabae disease severity on stems measured in chamber).

*, **, and *** significance levels at p< 0.05, p< 0.01, and p<0.001, respectively.

A)

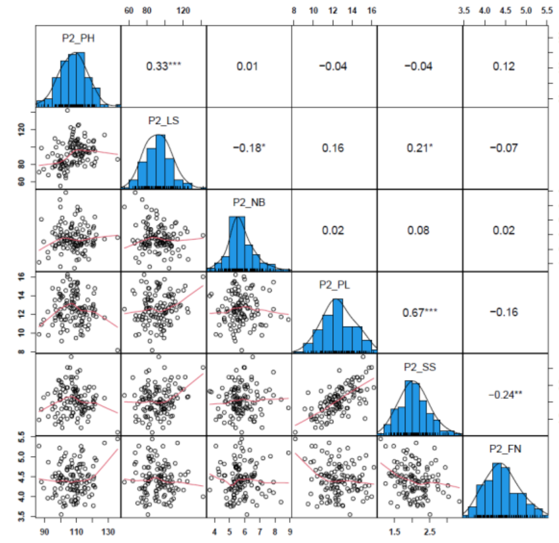

B)

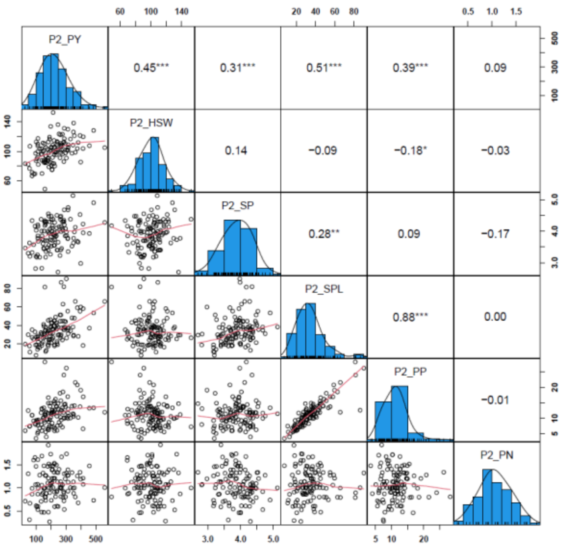

C)

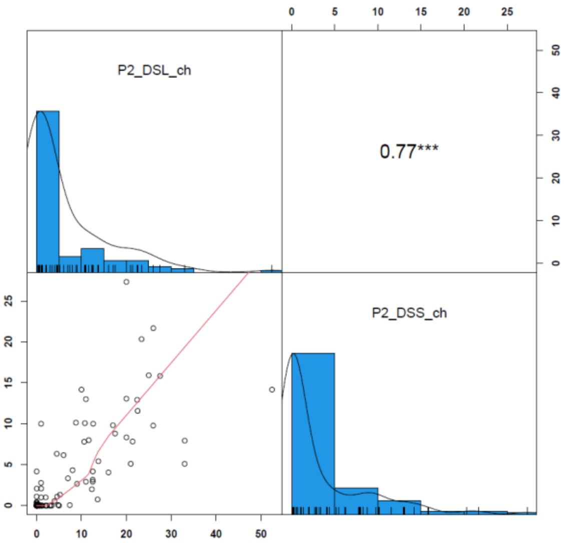

Supplement: Supplementary file 2 [file Image2.pdf]
