# Supplementary material for: Combining two high-density QTL maps with a reference genome to identify candidate genes for morphology, yield, and biotic resistance in faba bean (Vicia faba L.)
Source: Front Plant Sci. 2026 Apr 29;17:1832555. doi: 10.3389/fpls.2026.1832555 (PMC13167940; doi:10.3389/fpls.2026.1832555)

Supplementary Figure S4. Comparison between genetic and physical maps for P2 (Histal x L8). A) The collinearity between linkage groups (LG1.1/1.2-LG6) and corresponding physical chromosomes (Vf1S/L-Vf6). B) The correlations between genetic distances (cM) and physical positions (Mbp) for each linkage group.

A)

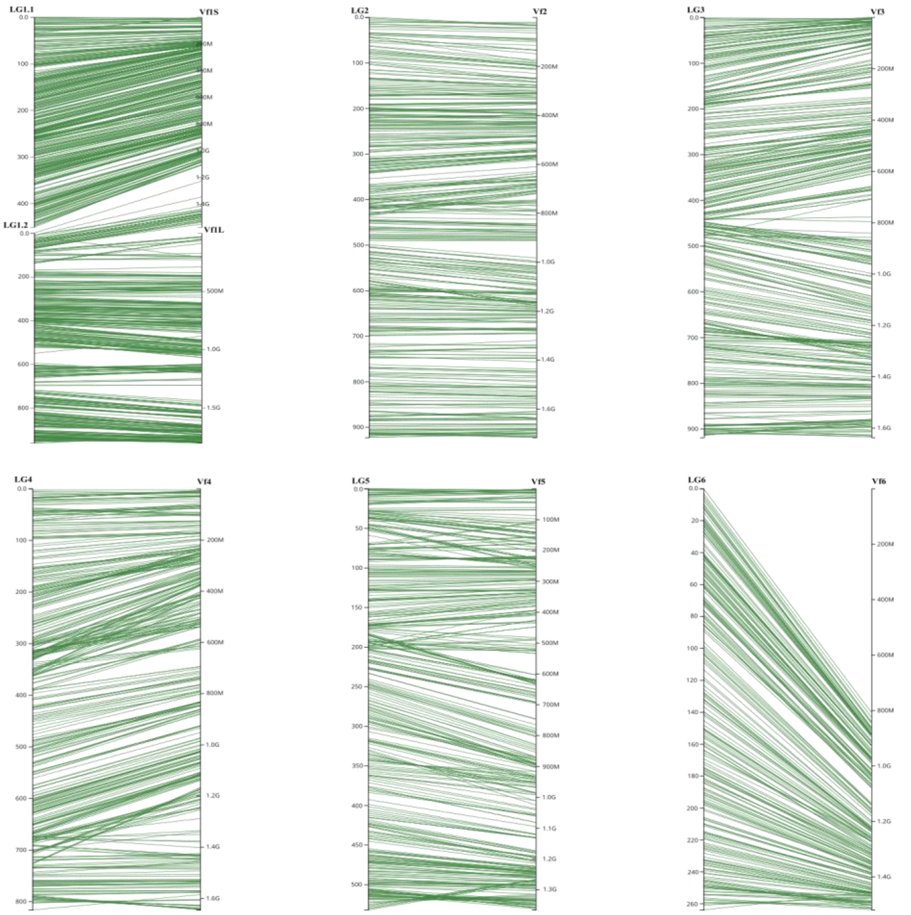

B)

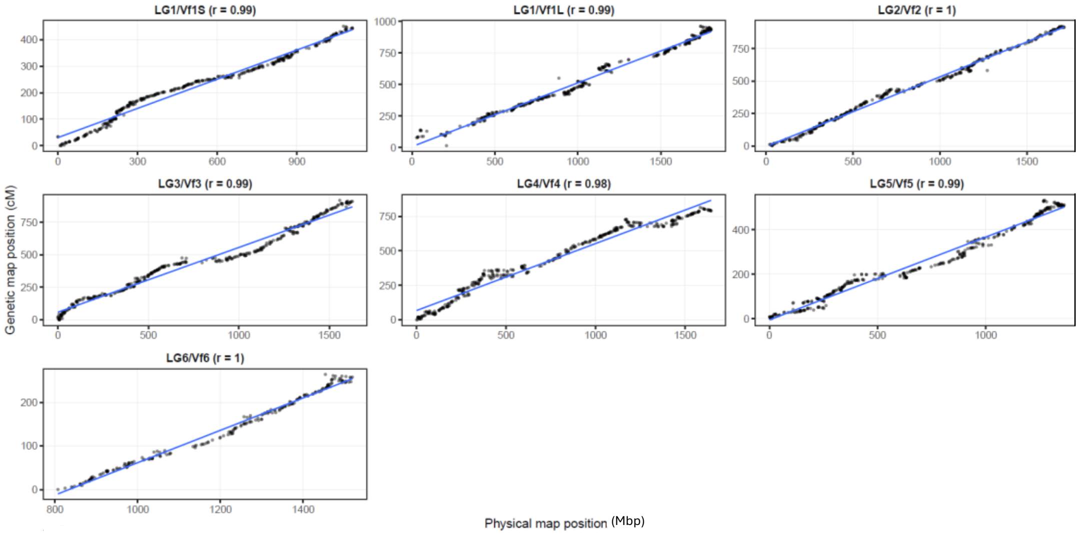

Supplement: Supplementary file 4 [file Image4.pdf]
