# Supplementary material for: Combining two high-density QTL maps with a reference genome to identify candidate genes for morphology, yield, and biotic resistance in faba bean (Vicia faba L.)
Source: Front Plant Sci. 2026 Apr 29;17:1832555. doi: 10.3389/fpls.2026.1832555 (PMC13167940; doi:10.3389/fpls.2026.1832555)

Supplementary Figure S1. Pearson correlations and histograms showing the distribution of traits in RIL population 1 (29H x Vf136). A) Phenotypic frequency and correlation between morphology traits: PH (plant height); LS (leaf size); NB (number of branches measured in the field); PL (pod length) and SS (seed size). B) Phenotypic frequency and correlation between yield-related traits: PY (plot yield); HSW (hundred seed weight); SP (seeds per pod measured in the field); SH (shattering); SPL (number of seeds per plant); PP (number of pods per plant). C) Phenotypic frequency and correlation between biotic resistance traits: DSL_ch (*Ascochyta fabae* disease severity on leaves measured in chamber); DSL_F (*A. fabae* disease severity on leaves measured in the field); DSS_ch (*A. fabae* disease severity on stems measured in chamber); DSS_F (*A. fabae* disease severity on stems measured in the field); DSP_F (*A. fabae* disease severity on pods measured in the field); IT (*A. fabae* infection type scale on leaves); Oc (*Orobanche crenata* resistance); Of (*Orobanche foetida* resistance).

*, **, and *** significance levels at $p < 0.05$, $p < 0.01$, and $p < 0.001$, respectively.

A)

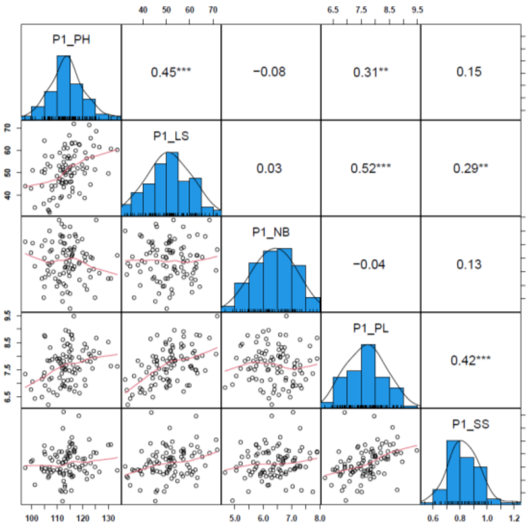

B)

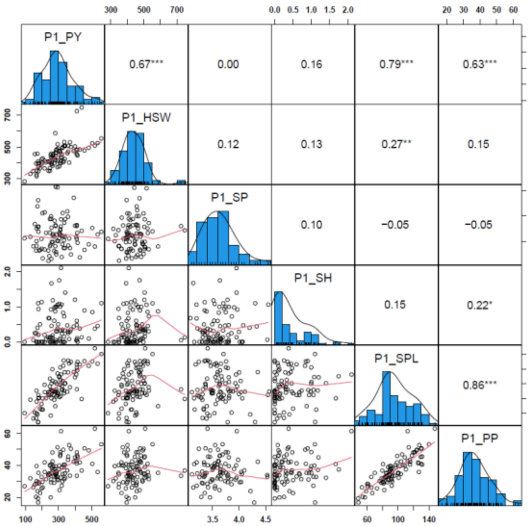

C)

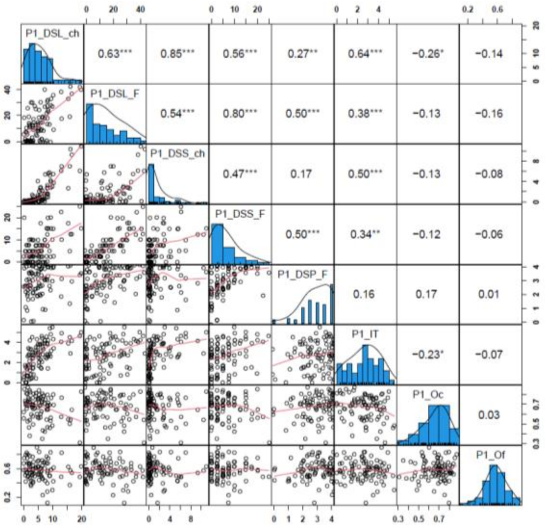

Supplement: Supplementary file 1 [file Image1.pdf]
